# Degenerate time-dependent network dynamics anticipate seizures in human epileptic brain

**DOI:** 10.1371/journal.pbio.2002580

**Published:** 2018-04-05

**Authors:** Adrià Tauste Campo, Alessandro Principe, Miguel Ley, Rodrigo Rocamora, Gustavo Deco

**Affiliations:** 1 Center for Brain and Cognition, Department of Information and Communication Technologies, Universitat Pompeu Fabra, Barcelona, Spain; 2 Epilepsy Unit, Department of Neurology, Hospital del Mar-Hospital del Mar Medical Research Institute (IMIM), Barcelona, Spain; 3 Faculty of Health and Life Sciences, Universitat Pompeu Fabra, Barcelona, Spain; 4 Institució Catalana de Recerca i Estudis Avançats (ICREA), Barcelona, Spain; ICM - Institut du Cerveau et de la Moelle épinière, France

## Abstract

Epileptic seizures are known to follow specific changes in brain dynamics. While some algorithms can nowadays robustly detect these changes, a clear understanding of the mechanism by which these alterations occur and generate seizures is still lacking. Here, we provide crossvalidated evidence that such changes are initiated by an alteration of physiological network state dynamics. Specifically, our analysis of long intracranial electroencephalography (iEEG) recordings from a group of 10 patients identifies a critical phase of a few hours in which time-dependent network states become less variable ("degenerate"), and this phase is followed by a global functional connectivity reduction before seizure onset. This critical phase is characterized by an abnormal occurrence of highly correlated network instances and is shown to be particularly associated with the activity of the resected regions in patients with validated postsurgical outcome. Our approach characterizes preseizure network dynamics as a cascade of 2 sequential events providing new insights into seizure prediction and control.

## Author summary

Understanding and predicting the generation of seizures in epileptic patients is fundamental to improving the quality of life of the more than 1% of the world population who suffer from this illness. Although seizure prediction has made important advances over the last decade, there is a lack of understanding of the common principles explaining the transitions that brain activity undergoes before a seizure. In this study, we characterized this transition from a novel perspective grounded on the mathematical analysis of continuous recordings inside the brains of epileptic patients over several days using depth electrodes. We show that the critical period preceding a seizure unfolds in a two-stage process. It begins with a phase of several hours when the highly correlated activity in the preceding days is altered, and it proceeds with a second, shorter phase of decrease in global connectivity before the seizure onset. Furthermore, our analysis reveals that these global alterations are more locally manifested in areas that are selected for surgical treatment. Our study suggests that preseizure activity might follow global stereotyped dynamics that could be targeted and modulated to prevent epileptic seizures.

## Introduction

Epilepsy is among the most common neurological disorders, with an estimated prevalence of about 1% of the world’s population and almost 2% in low-income families in developed countries [1]. Epilepsy is characterized by the seemingly random occurrence of seizures, which can greatly affect the quality of life of patients. Approximately one-third of all epileptic patients are resistant to pharmacotherapy [2] and could benefit from a variety of surgical options. Among them, closed-loop neuromodulation based on an accurate prediction of seizure occurrences is a promising tool.

Over the last decades, several studies have shown that seizures are preceded by detectable changes in brain dynamics that can be measured via intracranial recordings. Although not fully understood, these changes have been associated with the existence of a transition of interictal (period between seizures) activity into the preictal state [3,4]. These findings have motivated intense research on the development of seizure prediction algorithms for therapeutic use in patients with pharmacoresistant epilepsy [5–8]. Although significant progress has been made to attain above-chance level performance results [9], there is yet a long road to turn seizure prediction into therapeutic devices [8,10]. A major caveat of current seizure prediction is the lack of understanding about the neurophysiological processes associated with the emergence and maintenance of the preictal state. Indeed, most studies have resorted to fully data-driven methods to discriminate the preictal state with multiple signal features, which are typically patient specific and difficult to interpret [8].

Nowadays, epilepsy research is gradually adopting a network approach to study seizure dynamics at a global level and assess the contribution of the epileptogenic zone [11–14]. In this growing field, most studies have identified specific graph theoretical properties of functional networks during ictal and interictal periods [15–18]. In particular, a few groups have started to characterize the temporal variability of such functional networks during ictal [19–21] and interictal epochs [22–24]. Specifically, some authors have employed state spaces to classify recurrent functional networks during seizures to pinpoint those states that were responsible for the generation, maintenance, and termination of ictal activity [20,21]. More recently, a similar approach has been applied to a large sample of 10-min interictal epochs showing that interictal activity exhibits larger fluctuations than ictal periods over a common set of states [24]. In this context, however, the crucial question on whether there exist network dynamics changes pointing towards an upcoming seizure remains unaddressed. It is therefore due to ask: how are recurrent network states dynamically altered before epileptic seizures? And more generally, can network dynamics provide a common principle of the preictal state?

In the current study, we addressed these questions for the first time by analyzing time-dependent alterations in the dynamic repertoire of the functional connectivity [25] during long continuous periods preceding seizures. Based on insights from other models [26,27] and recent findings showing network dynamics alterations between interictal and ictal epochs [24], we hypothesized that the variability of physiological (nondysfunctional) network states was reduced as interictal activity approached epileptic seizures. Under this hypothesis, we developed a novel analysis to study specific variability changes prior to seizures preceded by long interictal periods in 10 epileptic patients monitored with intracranial electroencephalography (iEEG) during presurgical diagnosis. We made use of a graph theoretical property—the eigenvector centrality—to characterize network states [20] as instances of a time-varying multivariate continuous variable and resorted to the Gaussian entropy [28] to describe their variability. A controlled analysis using time-matched periods of interictal activity from additional days revealed a consistent and sustained decrease of the variability of network states before the seizure occurred. Remarkably, in all patients, this loss of variability was specifically associated with an abnormal occurrence of high-connectivity states (HCSs) during the preseizure period. We also investigated the contribution of the epileptogenic sites to the measured effect in 2 patients with long-lasting (>4 y) very good postsurgical outcome. In particular, the application of our analysis to the mapped epileptogenic sites of these seizure-free patients showed a significant alteration in the resected areas of the patients’ epileptic networks. Overall, our approach provides 2 main contributions in the analysis of epileptic network dynamics. First, it characterizes the preictal state as a 2-stage process in which epileptic networks undergo a functional reorganization before seizure onset. Second, it develops methodological aspects that may be considered to improve seizure prediction algorithms. More broadly, the results presented here open new lines to investigate dynamic alterations in pathological networks by studying the time-varying nature of brain activity.

## Results

**Fig 1:**
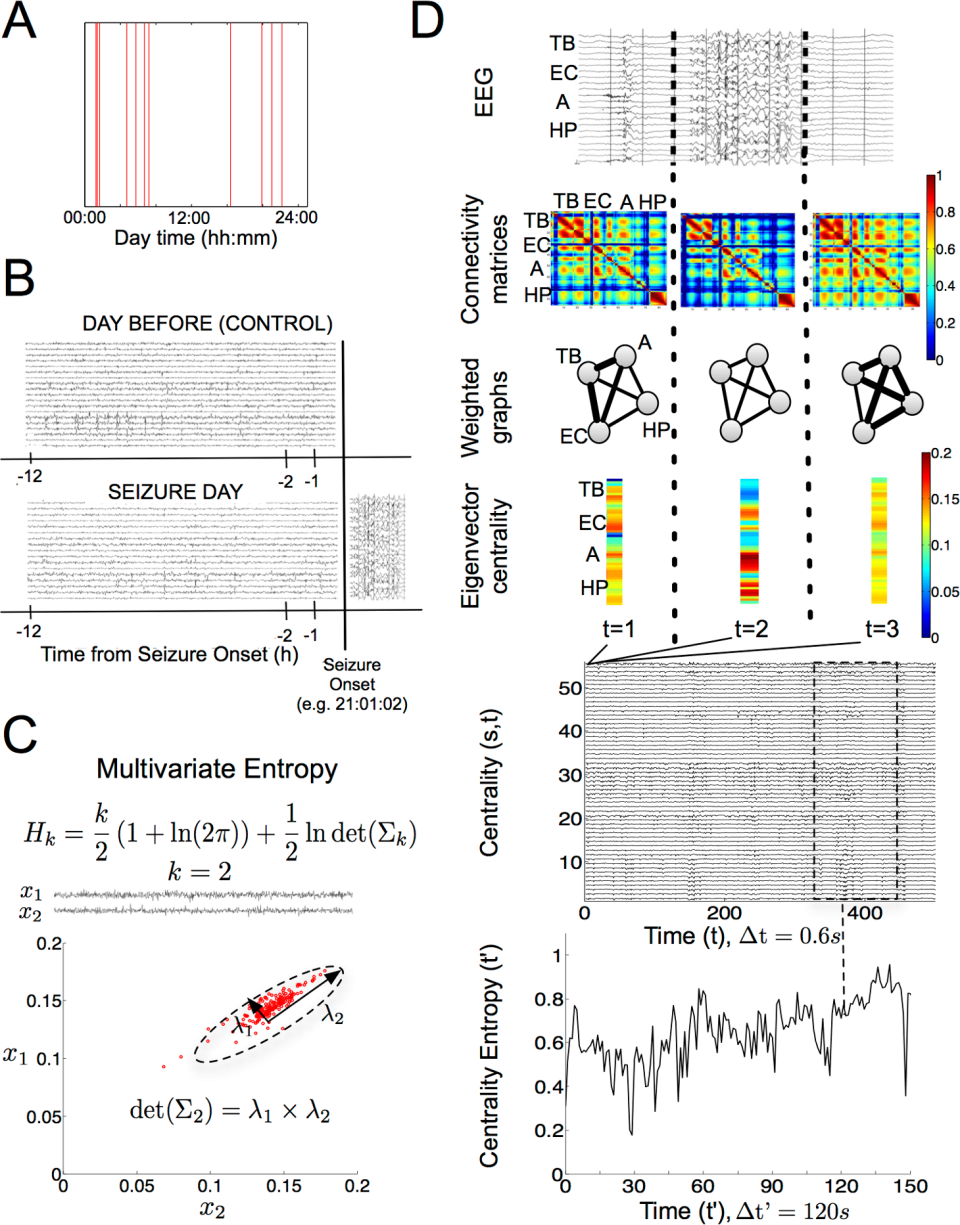
Study paradigm and network dynamics analysis. (A) Seizure onset time of the first recorded spontaneous clinical seizure from every patient (*n* = 10). (B) Schematic representation of the experimental design: for each patient, a preseizure period of up to 12 h was matched to the same time period of the previous day that served as a baseline reference (control interictal period). (C) Multivariate (Gaussian) entropy, showing its dependence on the determinant of the covariance matrix. Example for a case of 2 time series in which the determinant of the covariance is shown to shape the joint variability. (D) Network dynamics analysis: simultaneous intracranial EEG recordings were first divided into consecutive and nonoverlapping time windows of 0.6 s (top). Then, functional connectivity matrices were computed using zero-lagged absolute-valued Pearson correlation in each time window (middle-top 1). These matrices were modeled as weighted undirected graphs such that nodes represented recorded contacts and edges strength represented correlation absolute values (middle-top 2). The centrality of each contact in every graph was evaluated using the eigenvector centrality leading to a sequence of centrality vectors (middle-bottom 1). The overall eigenvector centrality sequence was regarded as a set of simultaneous centrality time series with 1 time series per recording site, over time steps of 0.6 s (middle-bottom 2). Finally, time-dependent centrality entropy values were found for each period of interest by sequentially estimating the multivariate entropy of the centrality time series in consecutive and nonoverlapping time windows of 120 s (200 samples). The labels TB, EC, A, and HP are used as an example to illustrate where the anatomical information was conveyed in the initial steps of the analysis. A, Amygdala; EC, Entorhinal cortex; EEG, electroencephalography; HP, Hippocampus; TB, Temporal basal area.

We studied network dynamics prior to epileptic seizures in 10 drug-resistant patients using continuous multichannel intracranial recordings via video stereoelectroencephalography (SEEG) during presurgical monitoring evaluation (see details in Fig 1). To capture long-term changes in network dynamics, we considered patients whose first spontaneous clinical seizure occurred after at least 30 h (average value: 71.4 ± 19.1 h; mean ± SD) of intracranial implantation. This ictal activity exhibited variable onset times over patients who were more concentrated during the 0:00 to 8:00 period (Fig 1A). For every patient, we analyzed a long continuous period (average value: 10.4 ± 1.9 h; mean ± SD) of intracranial activity before the seizure occurred (preseizure period, Fig 1B). We controlled for the specificity of our findings by independently analyzing time-matched periods of interictal activity from different days (e.g., control period, Fig 1B). In this study, we separately analyzed 8 patients (patients 1–8, main patients) with no clinically relevant events before the first seizure and 2 patients who presented potential factors perturbing the preseizure period (patients 9 and 10, control patients) (Table 1). More precisely, patient 9 had been electrically stimulated 16.5 h before the first recorded seizure, and patient 10 presented a subclinical seizure 6.1 h before the first clinical seizure onset.

**Table 1:** Main data of patients included in the study.

Patient	Age/Sex	Recording time (h)	Epilepsy	Side	MRI	Electrodes (left)	Analyzed regions	Seizures	Epilepsy duration (y)	Surgery	Seizure outcome (Engel's class)	Follow-up period
1	32/F	24	TLE	R	Negative	5(0)	A, Ha, Hp, TP, Lateral OFC	FS w CA	11	ATL	IA	4 y
2	27/M	48	TLE	R	right amygdala enlargement	7(0)	A, Ha, Hp, TP, EC, Lateral OFC, TGi	FS w CA or FS wo CA	12	RFTC	IB	2.5 y
3	32/F	42.5	TLE	L	Negative	7(7)	A, Ha, Hp, TP, EC, Lateral OFC, PHCp	FS w CA or FS wo CA or FS w CA and tonico-clonic bilateral evolution	26	SAH	IB	4 y
4	40/M	45.45	PCE	L	reduced size of right hippocampus	10(8)	A, Ha (2), Hp, TP, EC, POC(2), W, AG	FS w CA or FS wo CA or FS w CA and tonico-clonic bilateral evolution	39	temporoparietooccipital resection	III	17 mo (Engel IA for 12 mo)
5	26/M	36.95	PCE	R	right hemispheric atropy	15(0)	TP, A, Ha, Hp, EC, POC (2), W, AG, Ia, Im, Ip, Lateral OFC, M1	FS w CA or FS w CA and tonico-clonic bilateral evolution	17	temporoparietooccipital resection	III	15 mo (Engel IA for 6 mo)
6	46/M	48	TLE	L	Negative	12(12)	A, Ha, Hp, TP, EC, iTG, Ia, Ip, TPCp, HS, FB, CGp	FS w CA or FS w CA and tonico-clonic bilateral evolution	37	RFTC	IA	19 mo
7	31/M	23.2	TLE	L	reduced size of left hippocampus	9(8)	A, Ha (2), Hp, TP, EC, W, PHCp, TOJ	FS w CA or FS w CA and tonico-clonic bilateral evolution	11	NO	_	_
8	24/M	44	TLE	R	right temporal polar blurring	15(0)	A, Ha, Hp, TP, EC, PHCp, W, B, TOJ (2), TGs, Lateral OFC (4)	FS w CA or FS w CA and tonico-clonic bilateral evolution	8	RFTC	III	16 mo
9	41/M	24	TLE	L	left temporal polar blurring	8(8)	A, Ha, Hp, TP, EC, Latreal OFC, PHCp, TOJ	FS w CA or FS wo CA or FS w CA and tonico-clonic bilateral evolution	39	RFTC	III	2.5 y
10	34/F	8.16	TLE	L	left posterior hippocampal lesion	10(9)	A, Ha (2), Hp, TP, EC, Lateral OFC, PHCp, TOJ, Im	FS w CA or FS w CA and tonico-clonic bilateral evolution	18	ATL	III	3 y

Abbreviations: A, amygdala; AG, angular gyrus; ATL, Anterior temporal lobectomy; B, Broca’s area; CA, consciousness alteration; CGp, posterior cingulate; EC, entorhinal cortex; F, female; FB, frontobasal area; FS, focal seizure; Ha, anterior hippocampus; Hp, posterior hippocampus; HS, Heschl’s area; Ia, anterior insula; Im, mid insula; Ip, posterior insula; L, left; Lateral OFC, lateral parts of the orbitofrontal cortex; M, male; M1, primary motor area; NO, not operated; PCE, posterior cortex epilepsy; PHCp = posterior parahippocampal cortex; POC = precuneus occipital cortex; R, right; RFTC, Radiofrequency thermocoagulation; SAH, Selective amygdalohyppocampectomy; TGi, inferior temporal gyrus; TGs, superior temporal gyrus; TLE, temporal lobe epilepsy; TOJ, temporal occipital junction; TP, temporal pole; TPCp, posterior temporoparietal cortex; w, with; W, Wernicke’s area; wo, without.

### Network dynamics analysis

We tracked network state dynamics for each patient separately over each recording session. To do so, we computed functional connectivity using Pearson correlation across all recording sites (also referred to as sites; average value: 98.3 ± 25.1 sites; mean ± SD) over consecutive and nonoverlapping time windows of 0.6 s (Fig 1D). Networks in each window were characterized as a weighted undirected graph, such that electrode contacts represented the nodes and absolute-valued pairwise correlations represented their weighted edges (Fig 1D). We then evaluated a centrality measure for each connectivity matrix to track network dynamics in a reduced and interpretable dimensionality space. Indeed, we computed the eigenvector centrality to reduce each *N* x *N* connectivity matrix to an *N*-dimensional vector, such that *N* was the total number of recording sites, thus obtaining a centrality sequence for each recording site (Fig 1D). This measure can be equivalently interpreted as the first principal component of the absolute-valued correlation matrix of the set of intracranial recordings in each window.

Our initial hypothesis was that the preictal state was associated with a reduction of physiological network states. We therefore tested this hypothesis by quantifying changes in the distribution of the eigenvector centrality sequences representing these network states. In particular, we assumed that the centrality time series could be approximated by a multivariate Gaussian distribution for a sufficiently large number of samples (*n* > 100) [29]. In principle, the second-order variability of a multivariate variable may exhibit 2 components: the temporal component, i.e., how the centrality of a recording site varies as a function of time, and the spatial component, i.e., how the centrality consistently varies across recording sites at a given time instance. A measure that simultaneously quantifies both components is the multivariate Gaussian entropy, which monotonically depends on the product of the covariance matrix’s eigenvalues (Fig 1C). This measure corresponds to the differential entropy of multivariate normally distributed variables [28], but it can be proved useful to approximate the variability of more general variables whose distribution is asymptotically Gaussian.

### Network state variability identifies time-dependent alterations before seizure onset

**Fig 2:**
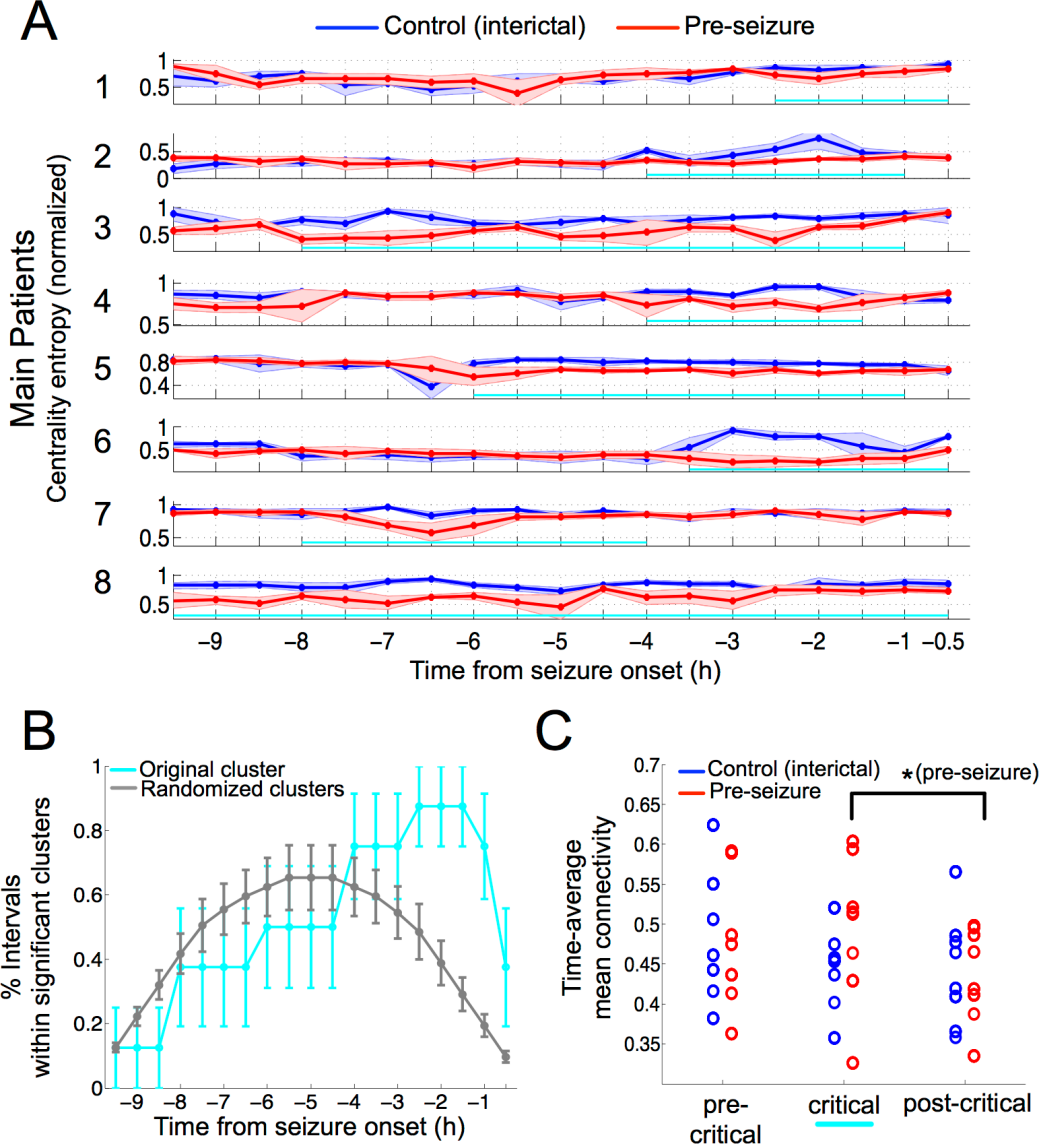
Time-dependent network state variability decreases near seizure onset during preseizure periods. (A) Average normalized—to the (0, 1) range—centrality entropy for the main epileptic patients (*n* = 8) during a preseizure period (in red, 9.5 h before the first seizure) and a control period (in blue, 9.5 h from the preceding day). Averages were computed over time in nonoverlapping windows of 15 entropy samples each (total of 30 min) during both periods. Each entropy sample was computed in a smaller window of 200 subsamples (120 s). Curves represent the sequence of centrality entropy mean values, and error bars represent ±1 SD. In cyan, the sequence of consecutive time steps lying in a significant clusterized difference (cluster-based test, *P <* 0.01). (B) Percentage of times that 30-min intervals lie within a significant cluster. In cyan, significant clusters are located in their original position. In grey, significant clusters are randomly placed along the preseizure periods of each patient. Error bars represent ±SEM. (C) Time-average mean functional connectivity per patient (*n =* 8) along 3 consecutive subperiods of interest during preseizure and control periods. The first subperiod (precritical) comprises intervals prior to the significant cluster, the intermediate subperiod (critical) comprises intervals within the cluster, and the last subperiod (postcritical) comprises postcluster intervals. In patients 1, 6, and 8, for whom the critical phase was attached to the seizure onset, the last interval was considered to belong to the postcritical subperiod. Star denotes that there was a significant difference between the critical and the postcritical subperiods of the preseizure period (*P <* 0.02, Wilcoxon test). Underlying numerical values can be found in S1 Data.

First, we centered our analysis on the preseizure period and the time-matched period from the previous day (preseizure, control). Over both periods, we computed the multivariate Gaussian entropy in consecutive and nonoverlapping time windows of 200 centrality samples (120 s) and normalized the measure to lie within the interval (0, 1) per patient. We shall refer to this applied measure as centrality entropy in the remainder of the article. The straightforward application of the centrality entropy to both periods in the main patients showed that centrality sequences were generally less entropic during the preseizure period (see S1A Fig), showing a gradual increase and successive decrease of this crossperiod difference as the seizure onset approached. In order to localize this effect in a specific and significant time segment, we grouped consecutive entropy values into intervals and made use of a nonparametric test to identify the cluster of consecutive centrality entropy intervals that was significantly yielding the largest entropy decay per patient (Materials and methods). The results of this test are illustrated for the main patients in Fig 2A where average centrality entropy curves are plotted for the control (in blue) and preseizure period (in red) together with the identified significant time segment (in cyan) during the 9.5 h preceding the seizure. In each patient, this segment highlighted intervals in which the same centrality entropy reduction could not be achieved by shuffling the entropy values within each interval across the preseizure and control periods (*P <* 0.01; S1B Fig). Intriguingly, the pinpointed segment was rather patient specific, exhibiting offset times that were not generally attached to the seizure onset. However, when grouping samples across the main patients, significant intervals turned out to be regularly distributed around the proximity of the seizure onset, with the interval (−2.5, −1.5) being the most frequent (87.5%; Fig 2B). In particular, this distribution was statistically different (*P <* 0.01, Kolmogorov-Smirnov test) from a surrogate distribution obtained by randomly placing the same segments per patient in every possible location of the preseizure period (Fig 2C). In addition, relevant features of the significant segment such as the onset and offset times and the test’s statistic value were not correlated with the seizure onset time (S2B, S2C and S2D Fig). These findings corroborated that our analysis controlled for possible underlying circadian modulations of the iEEG data (S2A Fig). Finally, the results obtained in both control patients were rather different from each other (S3A Fig). In particular, the crossperiod difference measured in patient 9 was the least significant across all patients (S3B Fig), suggesting that the previous received electrical stimulation might have had an effect on the preseizure dynamics. In contrast, the occurrence of a subclinical seizure in patient 10 did not yield a quantitatively different significance effect. We analyzed the stability of the results over the main patients using a synchronization measure over a wide range of frequency bands and an alternative centrality measure (Materials and methods). The separate application of both measures unravelled similar trends with weaker statistical effects (S4 and S5 Figs). In conclusion, our initial findings suggested that significant and sustained reductions of network state variability over a precedent-day baseline could be related to a preictal state. Furthermore, this reduction in variability was statistically mapped to a patient-specific time subperiod per patient. This subperiod will be referred to in the following as the critical phase.

As observed earlier, the critical phase was not, in general, attached to the seizure onset of every patient. Therefore, how could the critical phase be related to earlier reported evidence on the preictal state? To address this question, we divided both recording sessions into the critical phase and subperiods immediately before (pre-) and after (post-) the critical phase (Figs 2C and S3C for control patients). For those patients with critical phases attached to the seizure onset (patients 1, 6, and 8), we considered the postcritical phase to comprise the last window time samples of the critical phase. In each subperiod, we evaluated the mean functional connectivity during both recording sessions. Fig 2C shows that the mean connectivity exhibited a nonsignificant increase during the critical phase of the preseizure period (Fig 2C, *P* > 0.2, paired Wilcoxon test, *n =* 7 patients). In contrast, when comparing the critical and the postcritical phases of the preseizure period, the mean connectivity decreased significantly over all patients (Fig 2C, *P <* 0.02, *n =* 8 patients) in concordance with previous works [4,30,31]. This result was validated at a single-patient level in 7 out of 8 main patients (S6 Fig). Importantly, the postcritical effect was not present during the control period (*P* > 0.5), suggesting that the global connectivity decrease was specific to the preseizure period and could be a consequence of the critical phase.

### Reduced network state variability spans across spatial and temporal domains

As introduced earlier, the centrality entropy quantified the (spatiotemporal) variability of simultaneous centrality sequences in a single scalar value. Then how was the variability reduction individually expressed along recording sites and along time samples? To answer this question, we repeated the previous nonparametric statistical analysis (Fig 2A) over both recording periods using the spatial and temporal versions of centrality entropy independently (Materials and methods). S7B Fig shows that the statistical effect was present in both dimensions for every patient, but it was not equally distributed over space and time in all cases (S7 and S8 Figs). In sum, the decrease of network state variability observed during the preseizure period was associated with the occurrence of more similar centrality values over time (less temporal variability), which in general exhibited more homogeneous centrality values across recording sites (less spatial variability).

### Altered occurrence of HCSs explains reduction of variability

**Fig 3:**
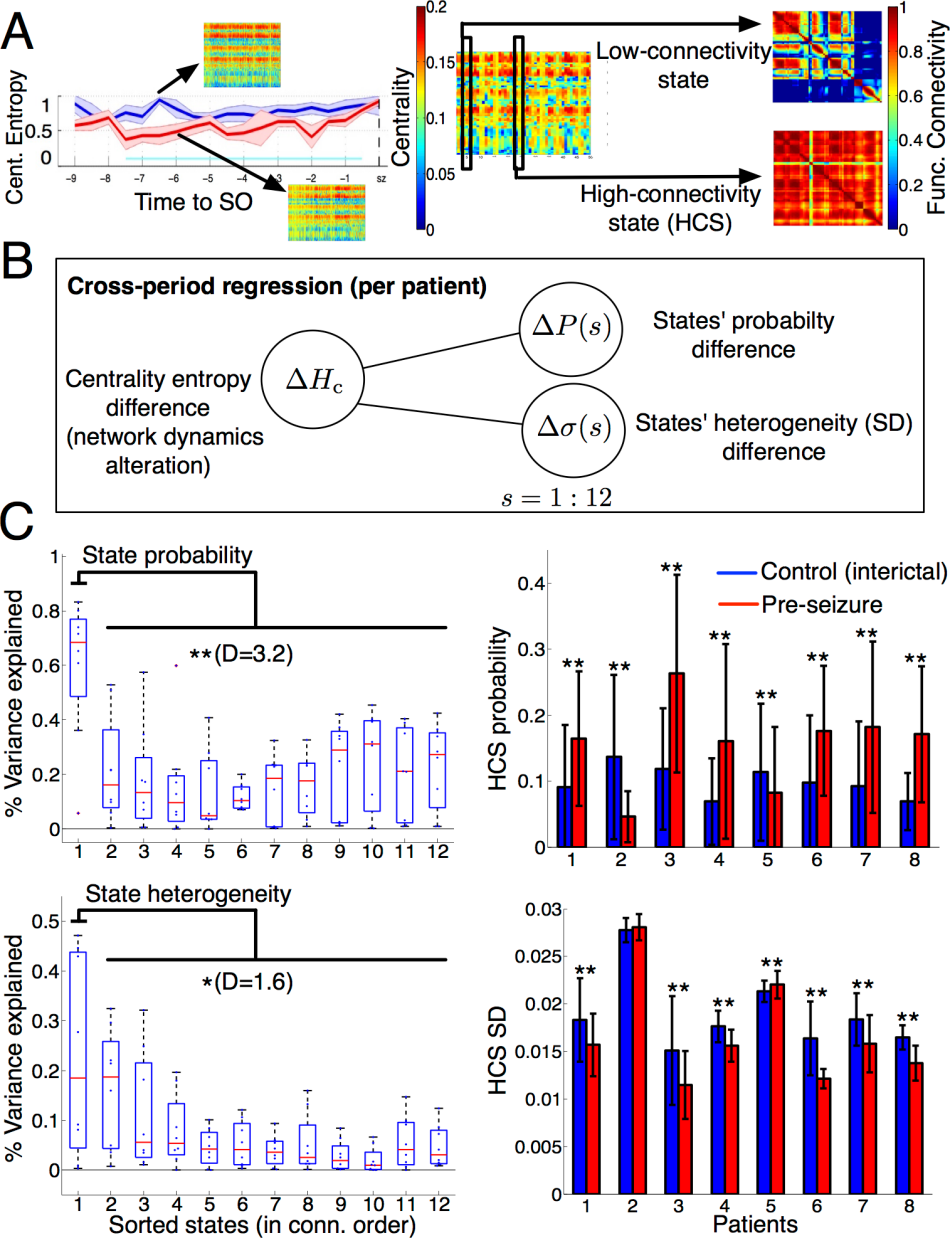
High-connectivity instances influence network dynamics alterations. (A) Inspection of centrality values around the critical phase (in cyan) suggested a higher presence of homogeneous (yellow strips) values across recording sites during the preseizure period (left), which were associated with high-connectivity correlation matrices (right). Color intensity (blue = lowest, red = highest) represents centrality and connectivity values across recording sites. (B) Schematic representation (1 per patient) of crossperiod entropy differences as a function of 2 families of regressors: changes of (discretized) states' probability and changes of states' heterogeneity across recording sites. (C) Variance explained by each family of regressors (top, state probabilities; bottom, state homogeneities) in every patient highlights HCSs as a common putative driver of the critical phase. Left: for each patient, discretized states (*n =* 12) were sorted along the horizontal axis in mean connectivity decreasing order. For each sorted state, boxplots show the distribution of the coefficient of determination (% variance explained) of each state across patients. Stars (* = *P <* 0.05, ** = *P <* 0.01, Wilcoxon test) denote the significance, and D (Cohen’s *d*) denotes the effect size of the difference between the coefficients of determination of HCSs and the remaining states. Right: crossperiod comparison per patient of regressor values associated with the HCS during the critical phase. Bars denote the average value of each regressor during the critical phase of the preseizure (red) and control (blue) periods per patient. Error bars denote 1 SD. Upper stars show that the differences in HCS probability and HCS SD were significant (* = *P <* 0.05, ** = *P <* 0.01, paired *t* test) after multiple-test correction. All variables in this regression analysis were computed in time windows of 200 time samples (120 s). Underlying numerical values can be found in S2 Data. HCS, high-connectivity state; SO, seizure onset.

The previous results described that network states (as modelled by the eigenvector centrality measure) became more temporally redundant and more spatially homogeneous during the critical phase. In turn, this reduced variability was associated with a nonsignificant variation of the mean connectivity across patients (Fig 2C). Yet what was the actual interplay between network dynamics and connectivity alterations during the preseizure period like? An initial time-varying analysis of the mean functional connectivity (averaged over all recording sites’ pairs) did not reveal consistent and sustained crossperiod differences over patients (S9 Fig). We then related the reduction in network variability to alterations in the occurrence of certain states. In particular, were there specific time-varying states producing the reported effect? We here explored this question and inspected the eigenvector centrality sequences during the control and preseizure periods. A visual inspection of these vector sequences for every patient suggested the hypothesis that the amount of “homogeneous states” (represented as yellow strips in the plot) was larger during the preseizure period than in the control period. Interestingly, these homogeneous states were specifically associated with high-connectivity correlation matrices in most of the patients (Fig 3A).

Centrality vector sequences like the one presented in Fig 3A were observed to be recurrent over time. Then, we used a clustering algorithm to extract the 12 most representative vectors over both periods of interest and classified each centrality vector at any given time accordingly (Materials and methods). Consequently, the sequence of centrality vectors turned into a sequence of discrete states whose frequency (or probability) over any time window could be computed and compared across control and preseizure periods. Then, we formally tested the hypothesis that the larger presence of homogeneous states during the preseizure period was associated with the observed reduction in network state variability in each patient. For each patient, we linearly regressed the crossperiod centrality entropy difference over 2 independent state regressors—state probability and state heterogeneity—the latter being measured as the SD across recording sites within a state (Fig 3B). We then computed the variance explained by each regressor via its coefficient of determination (R-squared). To investigate the group-level influence of every state’s connectivity into these associations, states were sorted for each patient in decreasing order of connectivity (i.e., mean connectivity of its associated absolute-valued correlation matrix), and coefficients of determination linked to state probability (Fig 3C, top) and state heterogeneity (Fig 3C, bottom) differences were distributed in boxplots for each state. Fig 3C (left) shows, for both regressors (state probability and state heterogeneity), that the most influential states on the reduced variability effect were those with highest-connectivity correlation matrices. Specifically, the difference between the variance explained by the highest-connectivity states and the remaining ones was significant in both state probability (*P <* 0.01, Wilcoxon test) and state heterogeneity (*P <* 0.05) with large effect sizes (D = 3.2, D = 1.6, Cohen’s *d*). Then, we computed the Spearman correlation between the highest-connectivity state regressors and the centrality entropy reduction across the main patients to unravel group-level correlation trends. Correlation values were of *r* = 0.7 (*P <* [1 × 10^−5^], n = 2328 time samples) and *r* = −0.45 (*P <* [1 × 10^−5^], n = 2328 time samples) for state probability and heterogeneity increases, respectively, indicating that the reduction of network variability was mostly explained by an increase in the frequency rate and homogeneity of the highest-connectivity states.

To further investigate the interplay of HCSs with the preseizure period, we evaluated crossperiod state probability and heterogeneity differences at the patient level during the critical phase previously identified in Fig 2A (Fig 3C right). First, we found that the probability of HCS was significantly different in all patients across both periods (paired *t* test, *P <* 0.01, multiple test–corrected, D > 0.5). In 6 out of 8 patients, HCSs occurred significantly more often during the critical phase, while they were less frequent in the 2 remaining patients (patients 2 and 5). Second, the homogeneity of HCSs were significantly increased in most of the patients (paired *t* test, *P <* 0.01, multiple test–corrected, D > 0.5), except in patient 5, for whom it significantly decreased, and in patient 2, for whom it remained statistically equal (*P* > 0.05). Although the influence of HCSs into the preseizure period was consistent across all patients, the differentiated trends found in some specific patients (patients 2 and 5) suggest that this influence might be modulated by context-dependent variables. In sum, HCSs strongly contributed to make state dynamics less variable over time by altering the overall states’ variability and imposing homogeneous centrality values across recording sites.

The key influence of HCSs into preseizure dynamics prompted us to evaluate the underlying traces of iEEG data during their corresponding time instances in periods of high- and low-centrality entropy. Our inspection of iEEG data from distinct epileptogenic sites over sequences of HCS and non–high-connectivity state (nHCS) instances (see S11 Fig for an example) identified these states as time segments in which the recorded electrical activity became transiently (low-centrality–entropy epoch) or more persistently (high-centrality–entropy epoch) synchronized. This synchronization was manifested through diverse patterns of oscillatory activity, which often included a slow wave. In parallel, a clinical evaluation by the epileptologists discarded any stereotyped epileptiform activity.

### Crossvalidation analysis in additional interictal periods

**Fig 4:**
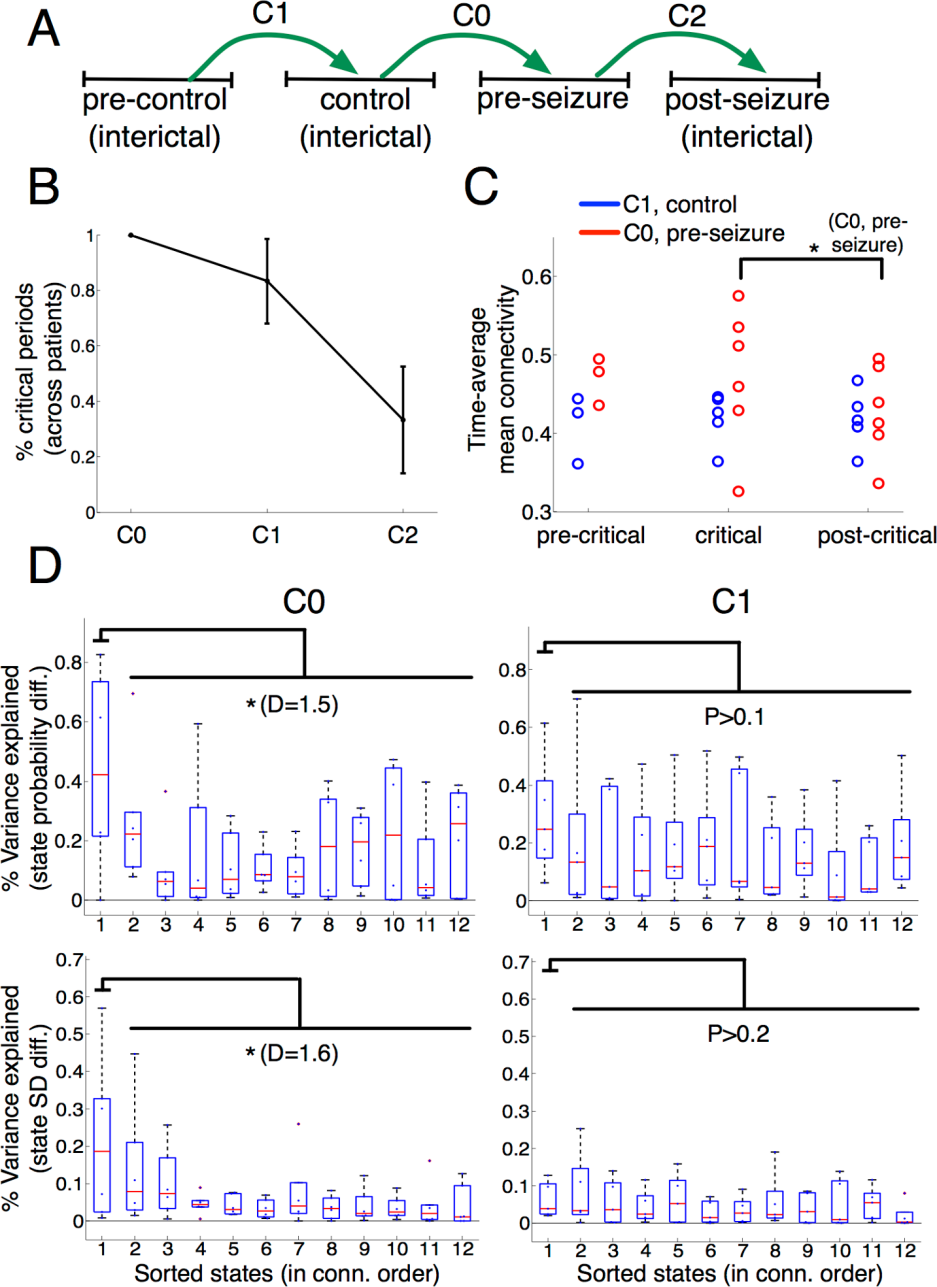
Crossvalidation analysis in additional periods (patients 2–6 and 8). (A) Schematic representation of the crossvalidation analysis involving patients 2–6 and 8 as well as 4 time-matched periods per patient. Time-matched periods from 2 d before the seizure (precontrol) and from a varying number of days after the seizure (postseizure) gave rise to 2 additional crossperiod comparisons (C1 and C2) to the previously analyzed comparison (C0). (B) Percentage of significant intervals across patients in the crossperiod comparisons C0, C1, and C2 (cluster-based test, *P <* 0.01). Error bars indicate SEM. (C) Reproducing the time-average mean connectivity plot of Fig 2C in comparisons C0 (in red) and C1 (in blue). The upper star indicates that the difference between the time-average mean connectivity values in the critical and postcritical phase trended a significant effect (* = *P <* 0.1, Wilcoxon test, *N* = 6) in C0. (D) Variance explained by each family of regressors of Fig 3B using the comparison C0 (left) and C1 (right). The upper star indicates that the difference between the coefficients of determination of HCSs and the remaining states trended a significant effect (* = *P <* 0.1, Wilcoxon test). D denotes the effect size (Cohen’s *d*) of this difference. Underlying numerical values can be found in S3 Data. HCS, high-connectivity state.

We identified network dynamics changes in the preseizure period that were consistently expressed with a similar trend (sustained variability reduction) across a heterogeneous cohort of patients (Fig 1). Critically, these time-dependent changes could be associated with a common factor in all patients, namely, an alteration of recurrent high-connectivity time instances (0.6 s) across recording sites. However, was this characterization specific to the preseizure period? Or could it be alternatively ascribed to a postimplantation effect? To shed light onto these questions, we analyzed an additional 121 h of interictal activity in 6 patients from time-matched periods that were placed 2 d before the seizure (“precontrol” period) and a varying number (across patients, mean = 3.83) of days after the seizure (“postcontrol” period). These new interictal data were introduced in the analysis as schematized in Fig 4A As control experiments, we defined 2 additional time-matched comparisons: a comparison between the precontrol and control periods (“C1”) and a comparison between the seizure and postseizure period (“C2”). These new comparisons were then confronted with the original comparison particularized to the 6 patients (“C0”). The overall analysis was made under the condition that period lengths were time matched and balanced across comparisons for each patient. First, for every comparison, we repeated the nonparametric statistical analysis of Fig 2A to determine the existence of putative critical phases in other periods. While comparison C2 only yielded 1 patient with a significant effect, C1 revealed that entropy reductions could also occur in non-preseizure periods in 5 out of 6 patients (Fig 4B). Nonetheless, when grouping the 6 patients in Fig 4C, the entropy reductions of C1 were not followed by a functional connectivity decrease, in contrast to C0, where the decrease showed a significant trend (*P <* 0.1, Wilcoxon test). Finally, we repeated the regression analysis of Fig 3B in patients with significant entropy reductions of C1 (5 patients) and C0 (original comparison in 6 patients) and represented the results analogously for each comparison. Crucially, for C1 periods, the variability decrease was more weakly explained by crossperiod HCS differences than in C0 periods. Indeed, the significant trend of C0 in the superior variance explained by HCSs (as compared to nHCSs) in both state probability (*P <* 0.1, D = 1.5) and state homogeneity (*P <* 0.1, D = 1.6) could not be reproduced by C1 (*P* > 0.1). In conclusion, although decreases in network state variability may occur across consecutive days (C1) preceding a seizure, we showed that those occurring during the preseizure period were specifically tied to HCS alterations and a subsequent functional connectivity decrease.

### Influence of the critical phase into epileptogenic sites

**Fig 5:**
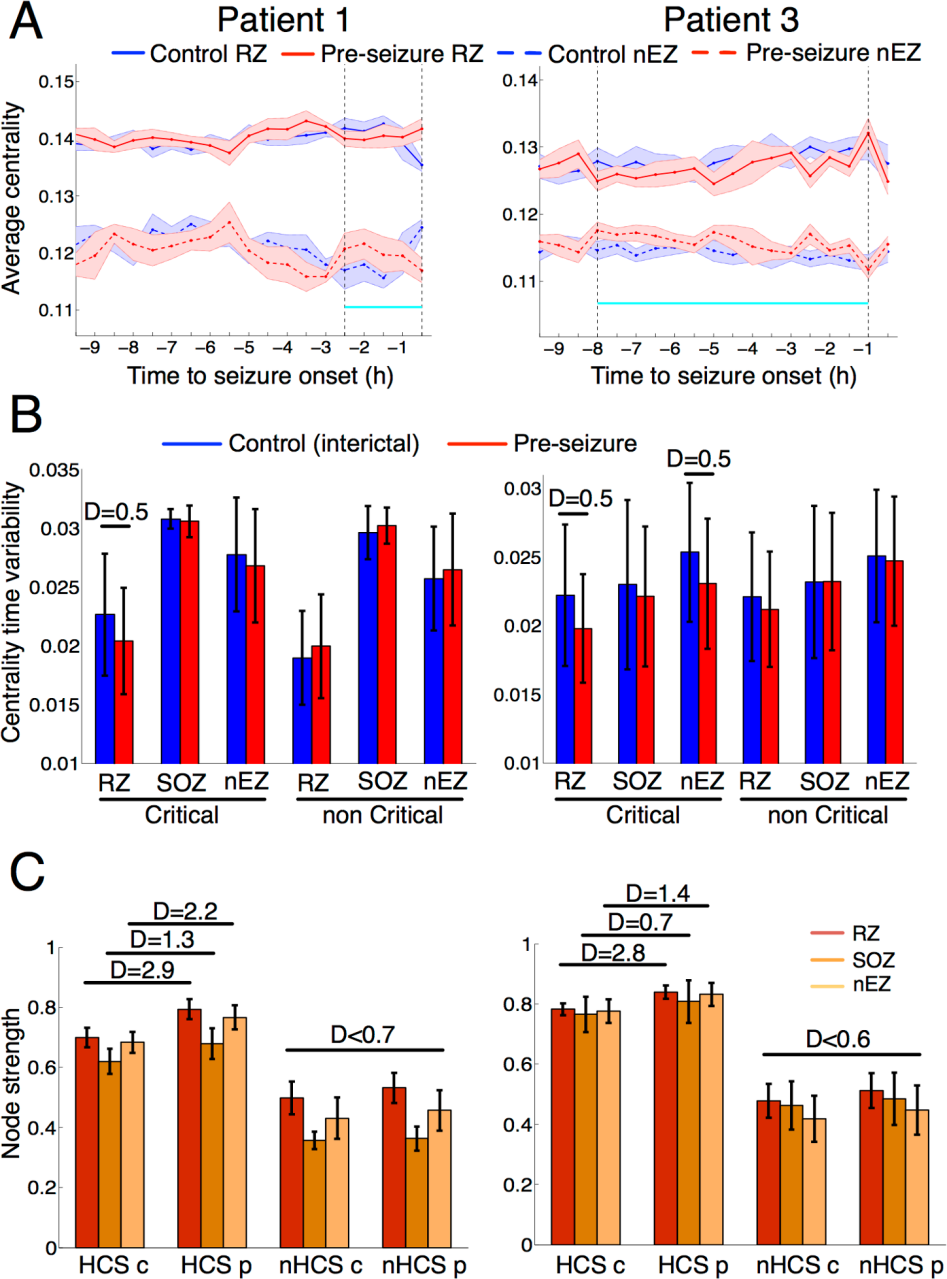
Epileptogenic sites are specifically altered in the critical phase (patients 1 and 3). In the 2 patients with the best postsurgical outcome after resectomy, recording sites in the RZ, SOZ, and in none of these regions (nEZ) were independently analyzed. (A) For each patient and period, site-average eigenvector centrality in the RZ and nEZ averaged within consecutive and nonoverlapping time windows of 120 s (200 samples) for 9.5 h prior to seizure-onset time. In solid lines, average centrality of the RZ. In dashed lines, average centrality of the nEZ. Blue and red curves stand for the control and preseizure periods, respectively. For illustration purposes, curves were averaged within windows of 30 min (15 samples per window) to enable direct comparison with the estimated critical phase (highlighted in cyan between 2 dashed vertical lines). Error bars denote 1 SD. (B) Crossperiod comparison (control in blue, preseizure in red) of sites’ centrality variability averaged over RZ, SOZ, and nEZ inside (critical, left) and outside (noncritical, right) the critical phase (cyan segment in panel A). Each sample per recording site was computed by performing an average of the centrality’s temporal SD measured in consecutive and nonoverlapping time windows of 120 s (200 samples). (C) Effect of HCSs into the epileptogenic zone. For each patient, bars showing the site-average connectivity strength RZ, SOZ, and nEZ during the HCS (outer left) and during the remaining states (nHCS, outer right) in control (inner left, “c”) and preseizure (inner right, “p”) periods within the critical phase. Strength samples were computed for each site by performing an average over all time instances (HCS and nHCS) during the critical phase. In (B) and (C), sizes of significant effects (paired *t* test, *P <* 0.05, multiple test–corrected) equal or larger to 0.5 were reported using Cohen’s *d* and approximated to the first decimal. In all subfigures, error bars represent ± 1 SD. Underlying numerical values can be found in S4 Data. c, control; HCS, high-connectivity state; nEZ, nonepileptogenic zone; nHCS, non–high-connectivity state; p, preseizure; RZ, resected zone; SOZ, seizure-onset zone.

Importantly, network dynamic changes observed during the preseizure period could be associated with an altered occurrence of HCSs in all patients. Yet how could this seemingly physiologic alteration evolve into generating seizures? In particular, how was this effect manifested in those regions that were involved in seizure generation? To relate our findings to the regional generation of seizures, we particularized our analysis to the clinically mapped epileptogenic sites of 2 patients with very good postsurgical outcome (Engel I) and a follow-up period of more than 4 y (patients 1 and 3, Fig 1, Materials and methods). Both patients are seizure free (Engel I), with patient 3 exhibiting some residual ictal symptomatology (seizure auras). In these patients, we specifically investigated the influence of epileptogenic sites in the preseizure network dynamic changes. To provide a complete comparison of sites, we independently analyzed seizure-onset zone (SOZ) sites (brain zone involved in the initial stages of the seizure spread), resected zone (RZ) sites (brain zone that rendered seizure-freeness after its resection), and the remaining sites (nonepileptogenic zone [nEZ] sites). In both patients, we note that the SOZ was not fully included in the RZ, and therefore the SOZ and RZ were partially overlapping regions. To carry out this region-specific analysis, we first evaluated the temporal mean and SD of the recording sites’ centrality in the SOZ, RZ, and nEZ sites over the control and preseizure periods. Fig 5A plots the time-average centrality of RZ and nEZ as a function of the remaining time to seizure onset. This figure illustrates in both patients that the time-average centrality of the RZ was higher than the nEZ over each period of interest, and—during the critical phase (in cyan)—the centrality of RZ sites was reduced at the expense of an increase in the centrality of nEZ sites. This preliminary observation suggested that both regions could participate in the preictal dynamics. However, was this participation equal across the 3 considered regions? Fig 5B characterizes the network dynamics of the 3 regions by comparing the temporal SD of their recording site’s centrality in SOZ (inner left), RZ (inner central), and nEZ (inner right) regions for control (blue) and preseizure (red) periods, inside (outer left) and outside (outer right) the critical phase. To assess crossperiod differences across regions of variable size, we highlighted significant differences (*P <* 0.05, paired *t* test, multiple test–corrected) exceeding an effect size threshold of 0.5 (large effect, Cohen’s *d*). Using this quantification, Fig 5B shows that the largest decrease in the centrality variability (D > 0.5) of patient 1 was only localized in the RZ during the critical phase. For patient 3, large effect sizes were found in RZ but also in nEZ during the critical phase. Outside the critical phase, crossperiod differences attained lower effect sizes (D < 0.3).

We next investigated the influence of HCSs on epileptogenic and nonepileptogenic sites to further describe the functional alterations occurring during the critical phase. More specifically, we compared the average connectivity per site (node strength) in the RZ, SOZ, and nEZ during the presence of the HCS and the remaining states (nHCS) in each patient for control and preseizure periods in the critical phase (Fig 5C). This analysis revealed several findings. First, in both patients, crossperiod differences in strength occurred more prominently during HCSs (average D > 1.8) than in nHCSs (average D < 0.7). Second, during HCSs, strengths increased from control to preseizure periods consistently in the 3 studied regions, while the differences were of varying signs across regions during nHCSs. Third, the region that exhibited the highest increase in strength was the RZ for both patients (D = 2.9, 2.8), followed by the nonepileptogenic sites (D = 2.2, 1.4) and the SOZ (D = 1.3, 0.7). Therefore, the abnormal occurrence of HCSs altered the connectivity gradient between epileptogenic and nonepileptogenic regions by strongly boosting the connectivity of the RZ sites. In particular, during the critical phase of the preseizure period, this increased connectivity was more persistent than in the control period, resulting in a reduced variability of RZ centrality values (Fig 5B). Finally, we evaluated how the postcritical functional connectivity decrease (Fig 2C) was spread over the 3 regions in both patients. S12 Fig shows that this effect was reproduced in each region (D ≥ 1.2), with, again, the RZ showing a more prominent decay (average D = 2.1).

To relate some of our regional findings with the patients’ postoperative outcome, we extended the analysis of the sites’ temporal variability (Fig 5B) to the main patients’ entire cohort (S13 Fig). This included 3 patients who underwent RFTC with variable outcomes (patients 2, 6, Engel I and patient 8, Engel III), 2 patients with bad postsurgical outcome after a follow-up period of more than 1 y (patients 4 and 5, Engel III), and 1 patient who was seizure free after SEEG monitoring (patient 7). The results are depicted for each patient in S13 Fig. Despite the variety of treatments and outcomes, S13 Fig consistently shows for all patients the larger contribution of epileptogenic sites than nonepileptogenic to the network variability change during the critical phase. Therefore, we elaborated on this observation in S4 Fig to show the temporal variability change of the nonresected and nonablated sites of all main patients during the critical phase as a function of their postoperative outcome. In general, the high values of the nontreated regions in Engel III patients (relative to Engel I patients) during the critical phase provide preliminary evidence that bad postoperative outcomes are associated with regions of large preseizure alterations not being treated.

## Discussion

**Fig 6:**
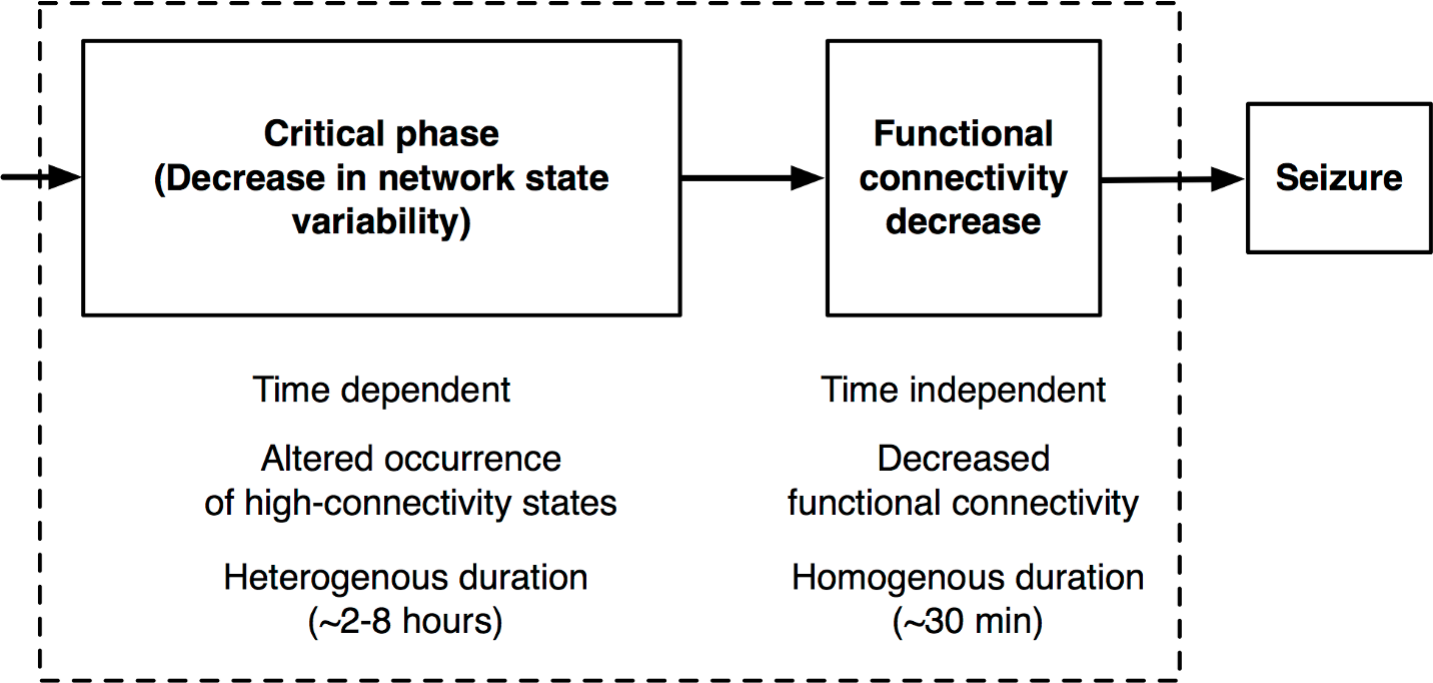
Scheme representing the preictal characterization with 2 sequential events of different nature and duration: The critical phase and the global functional connectivity decrease.

This study examined the existence of a common alteration principle in brain network dynamics during long-lasting periods of activity preceding the first clinical seizure in 10 patients with focal pharmacoresistant epilepsy. Using a comparative analysis between genuine preseizure periods and time-matched periods of interictal activity per patient, we were able to consistently show a sustained decrease in the variability of network states that was followed in most of the patients by a functional connectivity drop of approximately 30 min before the seizure onset (Fig 6). Further analysis revealed factors altering this variability in the temporal (time samples) and spatial (recording sites) domains. First, this decrease in network variability was associated with an abnormal occurrence of HCSs during preseizure periods as compared to previous days. Second, the reduction in temporal variability and the functional connectivity decrease was mainly localized in the RZ of the 2 patients with best postsurgical outcome.

Over the last decade, functional MRI (fMRI) studies have showed growing evidence that dynamic connectivity patterns (“brain dynamic repertoire”) may be an intrinsic property of brain function and disease [25]. Particular examples of disrupted dynamics have been found in Alzheimer disease [32] and neuropsychiatric disorders [33] for which translation to new clinical biomarkers is still a matter of discussion ([34] and references therein). In modern epilepsy research, the dynamic principle of brain function has been postulated to be commonplace to understand how seizures are generated [35], but most network studies have studied alterations in static functional network parameters, with a few recent exceptions [20,24,36–39]. In this context, our approach differs from previous works in several key elements. To name a few, we formulate a hypothesis about the variability of functional network states at short time scales (rather than using a grand-average measure), the analysis of long-lasting (approximately 10-h) continuous interictal periods (rather than a selection of short epochs), and—more importantly—the use of time-matched reference epochs outside the preseizure period to assess specificity. Next, we further elaborate on the latter point.

When studying the variability of brain dynamics along long recording periods, one is confronted with the confounding effect of circadian rhythms [23,36,40], which span across sleep and wake phases. These rhythms may become critical when one characterizes specific brain configurations associated with the preictal state, which has been shown to last approximately 4 h [41]. Previous studies on the preictal state have analyzed preictal changes with reference to previous interictal periods, not necessarily time-matched. Inspired by a previous work [42], the strategy used here tackled this issue by defining time-matched reference periods from precedent and subsequent days, thus allowing for a more specific identification of preictal changes in brain network dynamics. Although this approach may not be sufficient to control for all daily physiological state transitions, our preliminary data on the relationship between patients’ putative critical phases and seizure onset times discard the influence of daily rhythms into our main results. However, a larger cohort of patients with variable seizure times and a good readout of their sleep phases will be necessary to address this question in the future. Another key aspect of the study was the use of the first monitored clinical seizure occurring during the first implantation days. This choice was pivotal to analyze comparable long-term network dynamic changes across patients with limited influence of confounding factors such as the reduction of antiepileptic drugs, the effect of previous ictal processes, and the response to clinical stimulation. In most of the studied patients, this first seizure was the first event of a succession of seizures separated by short interictal periods of a few hours or minutes, which are clinically known as seizure clusters [43]. Therefore, understanding the preictal process of this initial seizure can also have important consequences for the control of later ictal activity. In any event, the analysis introduced here should be extended to subsequent seizures in future studies to determine whether the presented characterization is specific to seizures preceded by long interictal periods.

A central question in seizure prediction research has been the role of synchronization [44] during the preictal period. Some studies have reported drops in synchronization a few hours before seizure onset [30], while others have pinpointed the coexistence of distinct synchronization states depending on the recorded structures [12,31]. Even though a clear mechanism of such alterations is still missing, the most successful algorithms applied to large data sets make use of correlation matrices as key data features [9]. The findings presented in this study support the view that preictal correlation patterns are state dependent [22,24,31] over time windows of 600 ms, and therefore their alterations should be also analyzed and interpreted at this time scale. More precisely, our results suggest that a time-dependent variation in the occurrence of highly correlated time instances may be at the origin of the preictal state. This variation was manifested in most of the patients as an excess of HCSs, while in 2 patients it was manifested as a deficit. Although preictal connectivity trends are known to be patient specific [44], they should be further investigated against the influence of patient-dependent variables (e.g., implantation schemes, monitored behavioral states), a question that was outside of the scope of this study.

In recent years, there has been accumulated evidence that seizure generation and spread involves complex interactions between seizure-generating and surrounding areas [14,19,21]. Evaluating network dynamics in patients with good postsurgical outcome (>4 y), we were able to relate our findings to clinically mapped epileptogenic sites, namely the SOZ and the RZ, as well as the remaining sites. In these patients, the network centrality was higher in the epileptogenic than in nonepileptogenic sites, in line with previous studies [45,46,47]. Not surprisingly, changes in overall centrality within periods simultaneously occurred during the critical phase where centrality values from both regions approached (Fig 5A). Crucially, this occurred during a significant decrease in the (temporal) centrality variability of the RZ (Fig 5B), which was specific in patient 1 and also present in the nonepileptogenic sites of patient 3, who presented a slightly worse postsurgical outcome. The analysis of the influence of HCSs on validated epileptogenic sites supports the idea that these states might destabilize physiological state dynamics by increasing the connectivity of key sites within the epileptic network during the critical phase (Fig 5C). The follow-up of this phase is shown to be a global functional connectivity decrease, which is again more prominently manifested across resected nodes (S12 Fig). We speculate that this decrease in connectivity could be the result of central nodes of the epileptic network adopting a more autonomous activity that would result in the generation of a seizure. The inclusion in the analysis of additional patients with different postoperative outcome suggests that preseizure alterations in centrality variability may be a promising biomarker for targeting epileptogenic regions during surgery and ablation (S14 Fig). Yet a larger study including more seizure-free patients will be necessary to fully elucidate the mutual influence of physiological network dynamics and the epileptic network during the transition from interictal activity to focal seizures.

The results shown in this study prompt us to introduce new ingredients in seizure-prediction algorithms such as the control for daily rhythms [48] and the continuous tracking of time-dependent linear connectivity alterations at short time scales (<1 s). Some considerations are yet to be mentioned. First, the use of intracranial recordings is a limiting factor in the spatial analysis of brain states, thus making them a priori subject-dependent. Nonetheless, it is recognized that the SEEG methodology offers an optimal temporal and spatial resolution of neurophysiological recordings for neural signal analysis in comparison with other techniques in patients with epilepsy. Second, this study was aimed at defining network states in a linear and instantaneous form using zero-lagged functional connectivity rather than effective connectivity [49]. Although our results were validated against a nonlinear coupling measure at different narrow bands, the extension of our analysis to nonlinear [50] and linear [51] directional methods in follow-up studies may provide additional information on specific connectivity changes underlying preseizure alterations. In conclusion, this work provides electrophysiological evidence for characterizing the preseizure period as a long-lasting process in which epileptic networks undergo a sequential functional reorganization. Further investigations under this conception will help unravel seizure generation mechanisms from a network perspective, provide practical insights into how to predict and control ictal activity, and may constitute a general approach to analyze dynamic alterations of other neuropathologies.

## Materials and methods

### Ethics statement

All diagnostic and surgical procedures were approved by The Clinical Ethical Committee of Hospital del Mar, and all clinical investigation was conducted according to the principles expressed in the Declaration of Helsinki. Following the Declaration of Helsinki, patients were informed about the procedure, and they gave their written consent beforehand.

### Patients and recordings

A total number of 344 h of iEEG recordings from 10 patients with pharmacoresistant focal-onset seizures were analyzed. A summary of the patients’ characteristics is given in Table 1. We included patients who presented the first spontaneous clinical seizure in a timeframe that allowed us to perform a controlled analysis of EEG recordings during the preseizure period. Specifically, each patient in the study was selected if her first video SEEG–recorded clinical seizure had occurred after at least 30 h (average value: 71.4 ± 19.1 h; mean ± SD) with no presence of spontaneous clinical seizures. Among the selected patients, we included 2 patients presenting potential perturbation factors affecting the preseizure period (patients 9 and 10). Patient 9 had been electrically stimulated 16.5 h before the first recorded seizure, and patient 10 presented a subclinical seizure 6.1 h before the first clinical seizure onset.

For each patient, the selection of recording sessions was as follows. We considered up to 12 h before the first monitored clinical seizure occurred. As a baseline reference, we selected the same time period from the previous day (control period). For independent validation of our results, we selected additional time-matched periods of variable length in 6 patients (patients 2–6 and 8; average period length: 10 h) from 2 d before the seizure onset (precontrol period) and a few days after the seizure onset (postcontrol period; average value = 3.83 d). No more patients could be added to the validation analysis for time limitations (patients 7 and 10), a substantial modification of the implantation montage during the first monitoring days (patient 1), or the presence of direct electrical stimulation in the iEEG recordings (patient 9).

After detecting recording cuts in a few patients, we restricted the analysis to 11 h per session in patients 1 through 9 and to 2.4 h per recording session in patient 10 to ensure a time-matched crossperiod comparison. Among the selected patients, 2 patients achieved seizure freedom after surgical resection and radiofrequency thermocoagulation (RFTC, [52]) with a follow-up of 4 y and 3 y, respectively (patients 1 and 2, Engel IA). An additional patient only exhibited seizure auras after surgical resection and a follow-up of 4 y (patient 3, Engel IB). We considered patients 1 and 3 to have a validated very good postsurgical outcome. Therefore, for the purpose of analyzing epileptogenic sites, we separately considered the diagnosed SOZ and the RZ of these 2 patients in Fig 5. The SOZ was independently marked by 2 epileptologists (AP and RR) and consisted of *n =* 5 (anterior hippocampus) and *n =* 9 (anterior hippocampus, amygdala) recording sites for patient 1 and 3, respectively. The RZ covered 24 contacts in patient 1 (parts of anterior hippocampus, temporal pole, and entorhinal cortex) and 12 contacts in patient 3 (parts of anterior, posterior hippocampus, and amygdala). The remaining patients presented one of these cases: they had not undergone surgery (patients 2, 6, 8, 9), had a non–sufficiently long follow-up period (<18 mo, patients 4 and 5), had not yet been operated on (patient 7), or exhibited a bad postoperative outcome (patient 10). All recordings were performed using a standard clinical EEG system (XLTEK, subsidiary of Natus Medical, Pleasanton, CA) with a 500 Hz sampling rate. A uni- or bilateral implantation was performed accordingly, using 5 to 15 intracerebral electrodes (Dixi Médical, Besançon, France; diameter: 0.8 mm; 5 to 15 contacts, 2 mm long, 1.5 mm apart) that were stereotactically inserted using robotic guidance (ROSA, Medtech Surgical, New York, NY).

### Data preprocessing

Intracranial EEG signals were processed in the referential recording configuration (i.e., each signal was referred to a common reference). Examples of iEEG signals are displayed in S11 Fig. All recordings were filtered to remove the effect of the alternate current (Notch at 50 Hz and harmonics using a FIR filter). Then, signals were further band-pass filtered between 1 Hz and 150 Hz to remove slow drifts and aliasing effects, respectively. Artifacts were removed in each period by detecting time window samples (600 ms) such that mean (over pairs of sites) absolute-valued correlation values and mean (over sites) absolute-valued voltage amplitudes were 3 SDs larger than the median values across each period. To perform functional connectivity analysis, each iEEG signal was divided into consecutive and nonoverlapping 0.6 s–long windows (300 samples with 500 Hz sampling rate) to balance the requirements of approximate stationarity of the time series (requiring short epochs) and of sufficient data to allow accurate correlation estimates (requiring long epochs).

### Functional connectivity analysis

There are different methods to assess functional connectivity from time series data based on coupling measures [53,54]. Previous research on the comparison of linear and nonlinear coupling measures has resulted in having distinct “ideal” measures for distinct studied situations [55]. Here, we chose to employ Pearson correlation—a zero-lagged linear correlation measure—for its good tradeoff between simplicity and robustness [54] and, more importantly, because it allowed for a convenient definition of network state as it will be explained later.

Let *x* and *y* be 2 *N*-length time series representing 2 recorded signals and let $\overset{-}{x}$ and $\overset{-}{y}$ be their respective sample means. Their sample Pearson correlation is estimated as

$$r\left(x,y\right)=\frac{\sum_{i=1}^{N}(x\left(i\right)-\overset{-}{x})\left(y\left(i\right)-\overset{-}{y}\right)}{\sqrt{\sum_{i=1}^{N}{\left(x\left(i\right)-\overset{-}{x}\right)}^{2}}\sqrt{\sum_{i=1}^{N}{\left(y\left(i\right)-\overset{-}{y}\right)}^{2}}}$$ (1)

For each patient and each consecutive 0.6 s–long window, we computed the absolute value of the coupling measure across all pairs of electrode contacts. For most of the patients, the overall pairwise computations resulted in approximately 123,000 sequential connectivity matrices combining both recording sessions (control and preseizure periods). In the current study, we did not test the statistical significance of each pairwise coupling because our purpose was to track the overall network dynamics regardless of pairwise thresholding methods.

### Definition of network states

For each patient, we characterized each correlation matrix as a functional network. This network was modelled as a weighted undirected graph, such that electrode contacts represented the nodes and absolute-valued pairwise correlation values across represented their weighted edges [56]. Then, we computed the network measure of eigenvector centrality for each connectivity matrix [57]. For a given graph *G*=(*V*,*E*), let *A*=(*a*_*v*,*t*_) be its weighted adjacency matrix. The relative centrality score *x*_*v*_ of vertex *v* can be defined as

$$x_{v}=\frac{1}{\lambda }\sum_{t\in V}a_{v,t}x_{t},$$ (2)

which can be rearranged in a matrix form as *λx*=*Ax*. Given the requirement that all entries in *x* must be non-negative, the Perron–Frobenius theorem implies that only the greatest eigenvalue results in a proper centrality measure [57]. Therefore, the centrality measure is given by the eigenvector associated with the largest eigenvalue of the connectivity matrix. Then, the *i*th contact is assigned the *i*th component of this eigenvector such that *i* goes from 1 to number of recording sites in a patient. The eigenvector centrality is by definition a self-referential measure of centrality, i.e., nodes have high eigenvector centrality if they connect to other nodes that have high eigenvector centrality [58], which ultimately provides a measure of relative importance of each node in the network. The eigenvector centrality measure has been applied to resting-state fMRI studies [59] and more recently to ECoG recordings of epileptic patients [20].

By computing the centrality in each 0.6 s–long connectivity matrix, we obtained—for each patient—independent eigenvector centrality sequences along each recording session. If we consider each connectivity matrix to represent a brain state [60], these vectors can be regarded as representative elements of these states in a vector space of a dimension equal to the number of recording sites. Furthermore, these vectors point to the direction that best summarizes the original brain state. In particular, every time that a significant change arises in the connectivity matrix, the eigenvector centrality rotates to update the relative importance (“centrality”) of each contact within the new network configuration.

### Choice of zero-lagged correlation and eigenvector centrality

Computing the eigenvector centrality over zero-lagged connectivity matrices was key for regarding our network state measure as an informative summary of how the set of simultaneous iEEG recordings were instantaneously coupled within a short time window. Indeed, under these conditions, the eigenvector centrality corresponds, by definition, to the first principal component of the absolute-valued correlation matrix, i.e., the vector in the space of recording sites that accounts for the largest variance of the whole set of (normalized) iEEG recordings in a given time window. Combinations of other coupling measures and network features could lead to alternative definitions of network states. For the sake of comparison, we also provide in S4 Fig the results obtained by combining zero-lagged correlation with a different network feature—the node strength—which can be defined as the average pairwise connectivity of this node with the remaining ones [58]. Indeed, S4 Fig shows that the node strength yielded, in general, statistically weaker results than the eigenvector centrality. Furthermore, we investigated the possibility of combining a synchronization measure such as the phase-locking value [61] with the eigenvector centrality. This measure may capture contributions of non–zero-lagged couplings as well as nonlinear effects. To illustrate the difference between both measures in the frequency domain, we repeated the cluster-based statistical analysis of Fig 2A for consecutive frequency narrow bands over the range 1 to 120 Hz. S5 Fig shows that the results were qualitatively similar across all bands for most of the patients. Yet in those patients for whom discrepancies were found, the phase-locking value measure yielded weaker peaks than the (absolute-valued) zero-lagged correlation.

### Evaluating network state dynamics via Gaussian entropy

Our goal was to evaluate the variability of these representative states in each period. The long sequence of centrality vectors for each period can be equivalently regarded as a stream of simultaneous centrality time series, one for each recorded contact. Then, one can evaluate the spatiotemporal variability of the centrality time series through the application of the multivariate Gaussian entropy [28] in a given estimation time window, which we choose for this study to be 120 s. The multivariate Gaussian entropy is defined as

$$H_{c}=\frac{K}{2}(1+\ln\left(2\pi \right))+\frac{1}{2}\ln\left(det\;\Sigma \right),$$ (3)

such that *K* is the number of recording sites and *Σ* is the covariance matrix of the centrality time series estimated in the estimation windows. By considering centrality vectors to be independent, *Σ* in (4) becomes a diagonal matrix, and the Gaussian entropy captures the aggregated variability of the centrality vectors across the temporal dimension:

$$H_{c}{}^{\left(1\right)}=\frac{K}{2}(1+ln(2\pi ))+\frac{1}{2}\sum {}_{i=1:K}ln\;\Sigma {}_{i,i}.$$ (4)

By subtracting (4) from (3), one can evaluate the variability of the centrality vectors across the spatial dimension:

$$H_{c}^{\left(2\right)}=\frac{1}{2}\left(\ln(det\;\Sigma )-\sum_{i=1:K}\ln\;\Sigma_{i,i}\right).$$ (5)

Therefore, the 2 contributions sum up to give the Gaussian entropy (4):

$$H_{c}=H_{c}^{\left(1\right)}+H_{c}^{\left(2\right)}.$$ (6)

### Choice of window sizes (correlation and entropy)

The choice of 0.6 s (300 samples) for the correlation window was critical to gain statistical power. Choices of 1, 5, or 10 s were shown to weaken the detection of network dynamics changes because they were intermingling high- and low-connectivity effects in the same window. On the other hand, values of entropy windows ranging from 100 to 200 s yielded quite stable results. We selected a window size of 120 s (200 samples) because it offered a good tradeoff between estimation accuracy (200 samples are good enough to estimate covariance matrices of at most 120 variables) and stationarity.

### State clusterization

To associate the network variability decrease observed in the main patients with the occurrence of specific recurrent connectivity states, we jointly clustered the eigenvector centrality sequences in the analyzed time-matched comparisons using the k-means algorithm [62]. In the main results, we fixed the number of clusters to 12 to cover a sufficiently wide range of visually inspected connectivity states per patient. This cluster size was selected after exploring the stability of the results illustrated in Fig 3C for the range of values *n =* 8–12. In particular, S10 Fig shows that these results were qualitatively very similar for the choices *n =* 8, 10, 12.

### Statistical analysis

The preseizure decrease in centrality entropy was statistically tested as follows. We started by windowing consecutive entropy samples (*n =* 15, 30 min) in nonoverlapping and paired time segments across each period, and then we computed the effect size for each segment pair using Cohen’s *d* [63]. We then clustered adjacent segments with a criterion that effect size be larger than 0.15 (moderate effect) over a minimum of 4 adjacent segments (2 h) and considered the aggregated sum of these segments’ effect sizes as the main statistic. We further checked the statistical significance of this value through nonparametric statistical testing based on Monte Carlo sampling [64]. More concretely, for each patient with time segments satisfying the above criterion, we computed 1,000 random permutations of the centrality entropy samples across both conditions (within preseizure or control period) at each time segment and repeated the same segment clusterization procedure to obtain 1,000 surrogate statistic values. These values were used to approximate a null distribution against which we compared the original aggregated effect size value via a right-tail–sided significance test. If the test’s significance value was below 0.05, we considered the preseizure interval formed by the adjacent segments to exhibit significantly lower centrality entropy than the one obtained in the control period and we identified it as a critical phase. In addition, we made use of the Kolmogorov Smirnov test to assess that the critical phase distribution across patients was significantly different from a distribution of randomly placed significant clusters of the same duration.

In general, to test paired or unpaired samples across time (e.g., preseizure versus control period) or recording sites (e.g., seizure-onset sites across different periods) per patient, we made use of the Wilcoxon test for small sample sizes and the *t* test for sufficiently large numbers of samples (>30). However, in most comparisons, noncomparable or very large numbers of samples could overestimate statistical effects. Therefore, in those cases, we computed and reported the effect size using Cohen’s *d* (based on the difference between medians and/or means for Wilcoxon test and/or *t* test). To deal with the multiple-comparison problem, we applied the Holm–Bonferroni correction [65] over patients in Fig 3 and over combinations of regional comparisons in Fig 5. We resorted to linear regression and the coefficient of determination (R-squared) to evaluate the association between crossperiod differences in state probability and/or heterogeneity and the decrease in centrality entropy in Fig 3 and Fig 4. Finally, mean connectivity values across electrode pairs were computed using the Fisher transform [66].

### Note on the typology of statistical tests

The main results combined within-subject and group-level statistical tests depending on the question at hand. Within-subject tests can be found in Fig 2A, Fig 3C (right), and Fig 5. Group-level tests can be found in Figs 2B, 2C, 3C (left), 4B, 4C, and 4D.

## Supporting information

S1 Data Numerical values underlying Fig 2. Fig 2A results: Data that generate the centrality entropy plots and justifies the statistics of Fig 2A. Rows 2–9: patients 1–8; Columns 2–11: mean entropy during the control period, SD of the entropy during the control period, mean entropy during the preseizure period, SD of the entropy during the preseizure period, significant period indices, cluster-based test *P*-value, cluster-based statistic value, surrogates mean, surrogates SD, 1,000 surrogate vectors. Fig 2B results: top: percentage of intervals within the critical phase over patients for the original and surrogate distribution. Rows 2–3: original clusters, randomly placed clusters. Columns 2–3: percentage value, SEM. Bottom: *P*-value of the Kolmogorov–Smirnov test between both distributions. Fig 2C results: data that generate the mean connectivity plots across preseizure and control subperiods and justifies the statistics of Fig 2C. Top: rows 2–9: patients 1–8. Columns 2–7 in order: precritical subperiod during control period, precritical subperiod during preseizure period, critical subperiod during control period, critical subperiod during preseizure period, postcritical subperiod during control period, postcritical subperiod during preseizure period. Bottom: statistic results for the comparison across subperiod samples. Rows 12–15: *P*-value of paired Wilcoxon test in precritical versus critical (preseizure), *P*-value of paired Wilcoxon test in critical versus postcritical (preseizure), *P*-value of paired Wilcoxon test in precritical versus critical (control), *P*-value of paired Wilcoxon test in critical versus postcritical (control).
(MAT)

S2 Data Numerical values underlying Fig 3. Fig 3A results: data that generate the centrality entropy plots of patient 3. Fig 3C results: data that generate the boxplots of Fig 3C left and the bars of Fig 3C right, and justifies the statistics shown for each panel. Top: rows 2–9: patients 1–8. Columns 2–13: pairs of R-squared coefficients for states 1–12 (in decreasing mean connectivity value). Columns 14–25: mean HCS probability in control period, SD of HCS probability in control period, mean HCS probability in preseizure period, SD of HCS probability in preseizure period, *P*-value paired *t* test for HCS probability, effect size (Cohen’s *d*), mean HCS SD (heterogeneity) in control period, SD of HCS SD in control period, mean HCS SD in preseizure period, SD of HCS SD in preseizure period, *P*-value paired *t* test for HCS SD, effect size (Cohen’s *d*). Bottom: statistic results for the comparison between HCS and nHCS R-squared coefficients in probability and heterogeneity (Fig 3C left). Rows 12–13: HCS versus nHCS probabilities, HCS versus nHCS SD. Columns 2–3: *P*-value of paired Wilcoxon test, effect size of the test. HCS, high-connectivity state; nHCS, non–high-connectivity state.
(MAT)

S3 Data Numerical values underlying Fig 4. Fig 4B results: percentage (over patients) of significant periods found in each comparison. Rows 2–4: Comparison 0, Comparison 1, Comparison 2. Columns 2–3: percentage (over patients), SEM. Fig 4C results: data that generate the mean connectivity plots for Comparisons 0 and 1. Top: mean connectivity values for Comparison 0 (original) and Comparison 1. Rows 2–3: Comparison 0, Comparison 1. Columns 2–7: mean connectivity during precritical subperiod, SEM, mean connectivity during critical subperiod, SEM, mean connectivity during postcritical subperiod, SEM. Bottom: statistics of mean connectivity critical versus postcritical subperiod. Rows 6–7: Comparison 0, Comparison 1. Column 2: *P*-value paired Wilcoxon test. Fig 4D results: data that generate the boxplots of Fig 4D. Top: boxplots for comparison C0. Rows 3–8: patients 2–6 and 8. Columns 2–13: pairs of R-squared coefficients for states 1–12 (in decreasing mean connectivity value). Medium: Comparison C1. Rows 12–17: patients 2–6 and 8. Columns 2–13: R-squared coefficients for states 1–12 (in decreasing mean connectivity value). Bottom: statistic results for the comparison between HCS and nHCS R-squared coefficients in probability and heterogeneity. Rows 20–21: HCS versus nHCS probabilities, HCS versus nHCS SD. Columns 2–5: *P*-value paired Wilcoxon test for C0, effect size for C0 (Cohen’s *d*), *P*-value paired Wilcoxon test for C1, effect size for C1 (Cohen’s *d*). HCS, high-connectivity state; nHCS, non–high-connectivity state.
(MAT)

S4 Data Numerical values underlying Fig 5. Fig 5A results: mean eigenvector centrality values in the RZ and the nEZ. Rows 2–3: patient 1, patient 3. Columns 2–9: mean centrality in the RZ (control period), SD of the centrality in the RZ (control period), mean centrality in the RZ (preseizure period), SD of the centrality in the RZ (preseizure period), mean centrality in the nEZ (control period), SD of the centrality in the nEZ (control period), mean centrality in the nEZ (preseizure period), SD of the centrality in the nEZ (preseizure period). Fig 5B results: temporal variability in the RZ, SOZ, and nEZ. Top: differences in the critical phase. Bottom: differences in the noncritical phase. Rows 2–3 and rows 8–9: patient 1, patient 3. Columns 2–19: mean temporal variability in the RZ (control period), SD of the temporal variability in the RZ (control period), mean temporal variability in the RZ (preseizure period), SD of the temporal variability in the RZ (preseizure period), *P*-value paired *t* test, effect size (Cohen’s *d*), mean temporal variability in the SOZ (control period), SD of the temporal variability in the SOZ (control period), mean temporal variability in the SOZ (preseizure period), SD of the temporal variability in the SOZ (preseizure period), *P*-value paired *t* test, effect size (Cohen’s *d*), mean temporal variability in the nEZ (control period), SD of the temporal variability in the nEZ (control period), mean temporal variability in the nEZ (preseizure period), SD of the temporal variability in the nEZ (preseizure period), *P*-value paired *t* test, effect size (Cohen’s *d*). Fig 5C results: node strength in the RZ, SOZ, and nEZ. Top: node strength in HCS. Bottom: node strength in nHCS. Rows 2–3 and rows 8–9: patient 1, patient 3. Columns 2–19: mean node strength in the RZ (control period), SD of the node strength in the RZ (control period), mean node strength in the RZ (preseizure period), SD of the node strength in the RZ (preseizure period), *P*-value paired *t* test, effect size (Cohen’s *d*), mean node strength in the SOZ (control period), SD of the node strength in the SOZ (control period), mean node strength in the SOZ (preseizure period), SD of the node strength in the SOZ (preseizure period), *P*-value paired *t* test, effect size (Cohen’s *d*), mean node strength in the nEZ (control period), SD of the node strength in the nEZ (control period), mean node strength in the nEZ (preseizure period), SD of the node strength in the nEZ (preseizure period), *P*-value paired *t* test, effect size (Cohen’s *d*). nEZ, nonepileptogenic zone; RZ, resected zone; SOZ, seizure-onset zone.
(MAT)

S5 Data Numerical values underlying S1 Fig. (MAT)

S6 Data Numerical values underlying S2 Fig. (MAT)

S7 Data Numerical values underlying S3 Fig. (MAT)

S8 Data Numerical values underlying S4 Fig. (MAT)

S9 Data Numerical values underlying S5 Fig. (MAT)

S10 Data Numerical values underlying S6 Fig. (MAT)

S11 Data Numerical values underlying S7 Fig. (MAT)

S12 Data Numerical values underlying S8 Fig. (MAT)

S13 Data Numerical values underlying S9 Fig. (MAT)

S14 Data Numerical values underlying S10 Fig. (MAT)

S15 Data Numerical values underlying S11 Fig. (MAT)

S16 Data Numerical values underlying S12 Fig. (MAT)

S17 Data Numerical values underlying S13 Fig. (MAT)

S18 Data Numerical values underlying S14 Fig. (MAT)

S1 Fig Crossperiod differences of centrality entropy raw values (patients 1–8). (A) Centrality entropy curves for the control period (blue) and the preseizure period (red) are shown for all patients for 9.25 h preceding seizure onset time. In cyan, the sequence of consecutive time steps lying in a significant clusterized difference (randomization test, *P <* 0.01). (B) Results for the cluster-based significance test. White bars show the value of the cluster-based statistic. Grey bars show the average across all surrogate statistic values. Error bars indicate ± 1 SD. Underlying numerical values can be found in S5 Data.
(TIF)

S2 Fig Effect of daily rhythms. (A) Mean intracranial EEG signal energy (across channels and time windows of 120 s, bipolar montage) of patient 5 for more than 10 h preceding the seizure onset time in the control (blue) and preseizure period (red). The common time-dependent patterns of both traces reflect the potential effect of circadian rhythms into this basic measure. (B, C, D) Scatter plots of seizure onset time versus preictal segment properties (B, clusterized effect size; C, duration; D, remaining time to seizure onset) across patients 1–8. *P*-value lower bounds of Spearman correlation in each panel indicate that none of these associations was found significant. Underlying numerical values can be found in S6 Data. EEG, electroencephalogram.
(TIF)

S3 Fig Results for the control patients (patients 9 and 10). Time-dependent network state variability and functional connectivity for control patients. (A) Average normalized—to the (0, 1) range—centrality entropy for the control patients (*n =* 2) during a preseizure period (in red, 9.5 h before the first seizure) and a control period (in blue, 9.5 h from the preceding day). Averages were computed over time in nonoverlapping windows of 15 entropy samples each (total of 30 min) during both periods. Each entropy sample was computed in a smaller window of 200 subsamples (120 s). Curves represent the sequence of centrality entropy mean values, and error bars represent ± 1 SD. In cyan, the sequence of consecutive time steps lying in a significant clusterized difference (randomization test, *P <* 0.01). (B) Results for the cluster-based significance test. White bars show the value of the cluster-based statistic. Grey bars show the average across all surrogate statistic values. Error bars indicate ± 1 SD. (C) Time-average mean functional connectivity per patient (*n =* 2) along 3 consecutive subperiods of interest during preseizure and control periods. The first subperiod (precritical) comprises intervals prior to the significant cluster, the intermediate subperiod (critical) comprises intervals within the cluster, and the last subperiod (postcritical) comprises postcluster intervals. Underlying numerical values can be found in S7 Data.
(TIF)

S4 Fig Comparison between network measures. (A) Strength entropy curves for the control period (blue) and the preseizure period (red) are shown for every patient for 9.25 h preceding seizure onset time in a similar fashion as in Fig 2A. In cyan, the sequence of consecutive time steps lying in a significant clusterized difference (cluster-based randomization test, *P <* 0.01). (B) Results for the cluster-based significance test using the strength entropy (left) and the eigenvector centrality (right) to compute the multivariate entropies. White bars show the value of the cluster-based statistic. Grey bars show the average across all surrogate statistic values. Error bars indicate ± 1 SD. Underlying numerical values can be found in S8 Data.
(TIF)

S5 Fig Stability of the statistics of Fig 2A using a nonlinear measure and frequency narrow bands. Cluster-based statistic of the nonparametric test (Fig 2A) computed for eigenvector centrality sequences based on Pearson correlation (original, blue) and phase-locking value (red) on frequency narrow bands from 1 to 120 Hz. Underlying numerical values can be found in S9 Data.
(TIF)

S6 Fig Postcritical functional connectivity decrease per patient. Time-average mean functional connectivity computed for each patient in 3 periods of interest for 9.25 h preceding the seizure onset time: the precritical phase (1), the critical phase (2), and the postcritical phase (3). In patients 1, 5, and 8, the last interval of the critical phase was considered to be in (3). Patient 8 did not present any interval before the critical phase in the period considered here. Error bars denote the SD across time samples. Stars denote that the decrease was significant in 7 out of 8 patients (*P <* 0.01, paired *t* test). Underlying numerical values can be found in S10 Data.
(TIF)

S7 Fig Network state variability reduction across spatial and temporal dimensions. (A) Schematic representation of the 2 sources of variability in a set of simultaneous time series. (B) For every main patient, green dots representing pairs of statistic values (“temporal/spatial effects”) obtained from repeating the clusterized effect-size test (Fig 2A) with the spatial and temporal entropy, respectively. The circled effect pairs are exemplified in C. (C) For exemplary patients 2, 3, 5, and 8, the figure shows the decomposition of the centrality entropy values into pairs of temporal and spatial entropy values. In blue, pairs of entropy values obtained from the control segment. In red, pairs of entropy values obtained from the preseizure period. Underlying numerical values can be found in S11 Data.
(TIF)

S8 Fig Complementary information for Fig S6. Centrality entropy decomposition of patients 1, 4, 6, and 7 into pairs of temporal and spatial entropy values. In blue, pairs of entropy values obtained from the control segment. In red, pairs of entropy values obtained from the preseizure period. Underlying numerical values can be found in S12 Data.
(TIF)

S9 Fig Crossperiod comparison of mean connectivity. Time-average mean connectivity curves for the main patients for 9.25 h preceding seizure onset time. The mean connectivity was computed over all recording pairs in consecutive and nonoverlapping 0.6 s windows (300 samples). The time average was performed at the same time scale of the centrality entropy, i.e., 120 s (200 samples). In blue, curve corresponding to the control period. In red, curve corresponding to the preseizure period. Underlying numerical values can be found in S13 Data.
(TIF)

S10 Fig Effect of the number of discretized states into the results of Fig 3. Variance explained by state probability and state homogeneity (“state SD”) differences in the crossperiod regression per patient of Fig 3B. when using *n =* 8 (left), *n =* 10 (center) discretized states and the original discretization (*n =* 12) shown in Fig 3C. (left). In the 3 cases, discretized states were sorted along the horizontal axis in mean connectivity decreasing order for each patient. For each sorted state, boxplots show the distribution of the coefficient of determination (% variance explained) across patients. Underlying numerical values can be found in S14 Data.
(TIF)

S11 Fig Exemplary iEEG epileptogenic recordings in high- and low-centrality entropy periods (patient 3). (A) Sequence of 21 (12.6 s) consecutive centrality eigenvectors extracted from an epoch with high-centrality entropy values (left) and from an epoch with low-centrality entropy values (right) in the preseizure period of patient 3. Color intensity (blue = lowest, red = highest) represents centrality values. Horizontal dashed lines delimit HCSs (homogenous yellow strips) and nHCSs (heterogeneous strips) per epoch of 0.6 s duration. (B) Normalized and voltage-shifted (for visualization purposes) iEEG recordings in HA (channel 11, blue), HP (channel 24, red), and EC (channel 35, green) in the high-centrality entropy (left) and low-centrality entropy (right) epoch. Horizontal dashed lines delimit the iEEG recording segments corresponding to the previous HCSs and nHCSs. Underlying numerical values can be found in S15 Data. EC, entorhinal cortex; HA, anterior hippocampus; HCS, high-connectivity state; HP, posterior hippocampus; iEEG, intracranial electroencephalography; nHCS, non–high-connectivity state.
(TIF)

S12 Fig Postcritical global functional connectivity decrease across epileptogenic and nonepileptogenic sites. For patients 1 and 3 (patients with best postsurgical outcome after resection), bars showing the site-average connectivity strength of the RZ, SOZ, and remaining sites (nEZ) during the critical phase (left) and postcritical phase (right) in the preseizure period. Strength samples were computed for each site by performing averages over time samples (0.6 s) during each phase. Effect sizes were reported for each sites’ group by computing Cohen’s *d* across both phases. Underlying numerical values can be found in S16 Data. nEZ, nonepileptogenic zone; RZ, resected zone; SOZ, seizure-onset zone.
(TIF)

S13 Fig Extension of regional analysis to all main patients. For patients 2, 4, and 5–8, crossperiod comparison (control in blue, preseizure in red) of sites’ centrality variability averaged over RZ, SOZ, and nEZ inside (critical, left) and outside (noncritical, right) the estimated critical phase whenever it was possible. Each sample per recording site was computed by performing an average (across critical and noncritical phases) of the centrality’s temporal SD measured in consecutive and nonoverlapping time windows of 120 s (200 samples). Sizes of significant effects (paired *t* test, *P <* 0.05) equal or larger to 0.5 were reported using Cohen’s *d* and approximated to the first decimal. In all subfigures, error bars represent ± 1 SD. Underlying numerical values can be found in S17 Data. nEZ, nonepileptogenic zone; RZ, resected zone; SOZ, seizure-onset zone.
(TIF)

S14 Fig Relationship between centrality time variability of the nontreated zone and postoperative outcome. For the main patients (*n =* 7) who underwent either surgery or RFTC, dots represent the effect size (Cohen’s *d*) of preseizure changes in the centrality time variability of nontreated regions (nonresected or nonablated) as a function of the postoperative outcome. (A) Results for surgical treatment (patients 1 and 3–5) inside (left) and outside (right) the critical phase. (B) Results for RFTC treatment (patients 2, 6, and 7) inside the critical phase. Underlying numerical values can be found in S18 Data. RFTC, radiofrequency thermocoagulation.
(TIF)

## Abbreviations

fMRI: functional Magnetic Resonance Imaging
HCS: high-connectivity state
iEEG: intracranial electroencephalography
nEZ: nonepileptogenic zone
nHCS: non–high-connectivity state
RFTC: radiofrequency thermocoagulation
RZ: resected zone
SEEG: stereoelectroencephalography
SOZ: seizure-onset zone
