# Supplementary figures and images for: Degenerate time-dependent network dynamics anticipate seizures in human epileptic brain
Source: PLoS Biol. 2018 Apr 5;16(4):e2002580. doi: 10.1371/journal.pbio.2002580 (PMC5886392; doi:10.1371/journal.pbio.2002580)

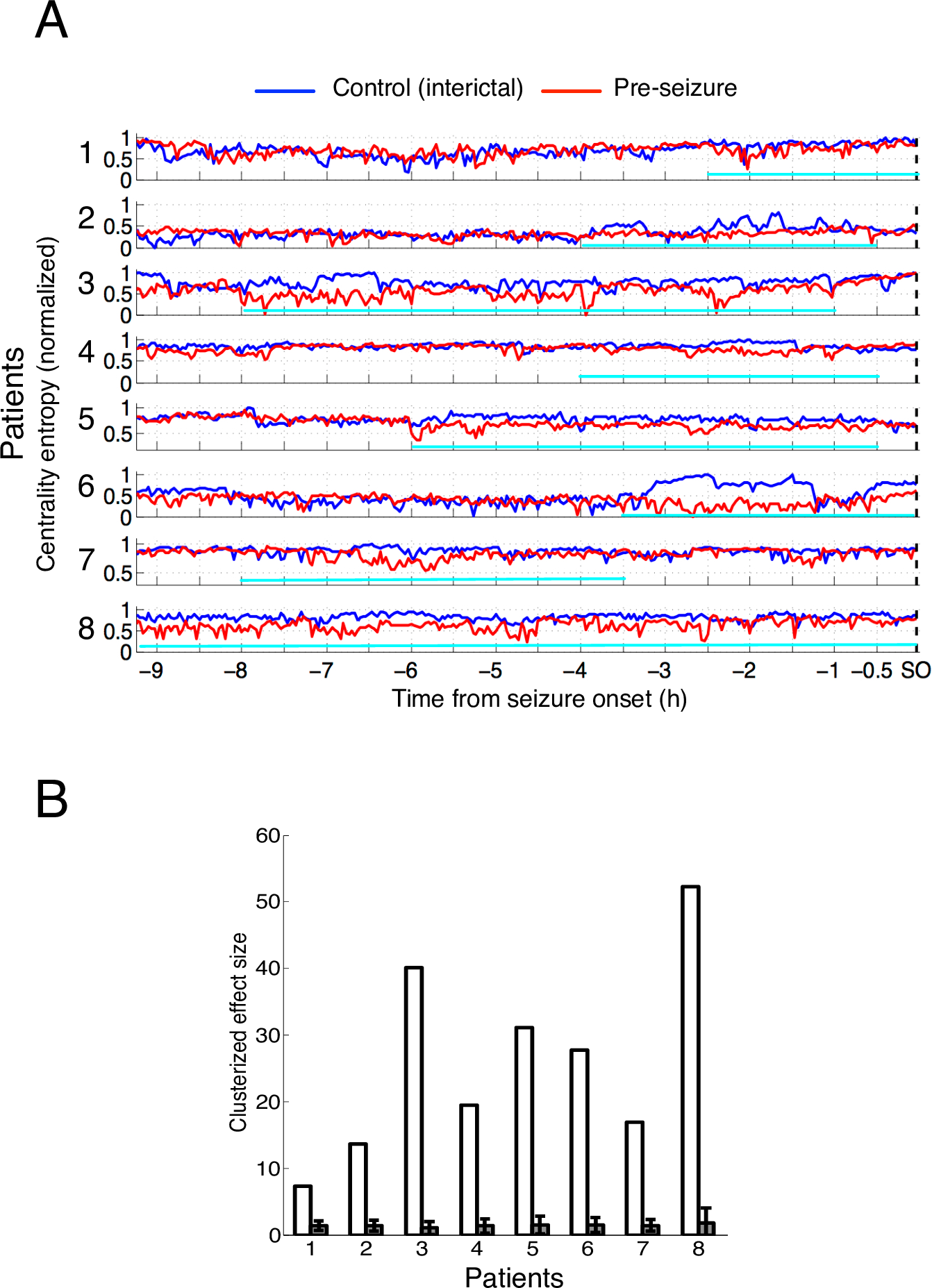

Supplement: S1 Fig — (A) Centrality entropy curves for the control period (blue) and the preseizure period (red) are shown for all patients for 9.25 h preceding seizure onset time. In cyan, the sequence of consecutive time steps lying in a significant clusterized difference (randomization test, P < 0.01). (B) Results for the cluster-based significance test. White bars show the value of the cluster-based statistic. Grey bars show the average across all surrogate statistic values. Error bars indicate ± 1 SD. Underlying numerical values can be found in S5 Data. (TIF) [file pbio.2002580.s019.tif]

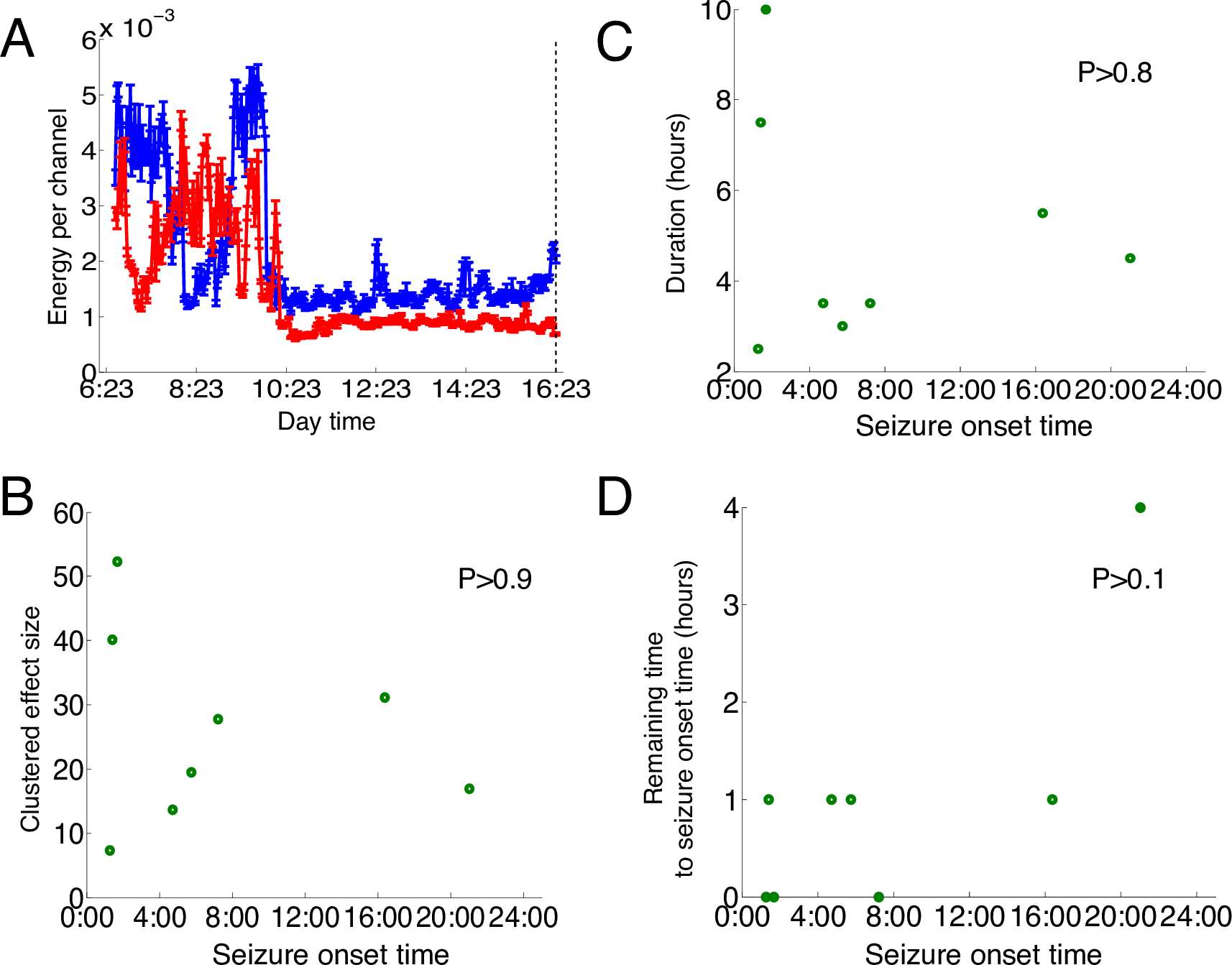

Supplement: S2 Fig — (A) Mean intracranial EEG signal energy (across channels and time windows of 120 s, bipolar montage) of patient 5 for more than 10 h preceding the seizure onset time in the control (blue) and preseizure period (red). The common time-dependent patterns of both traces reflect the potential effect of circadian rhythms into this basic measure. (B, C, D) Scatter plots of seizure onset time versus preictal segment properties (B, clusterized effect size; C, duration; D, remaining time to seizure onset) across patients 1–8. P-value lower bounds of Spearman correlation in each panel indicate that none of these associations was found significant. Underlying numerical values can be found in S6 Data. EEG, electroencephalogram. (TIF) [file pbio.2002580.s020.tif]

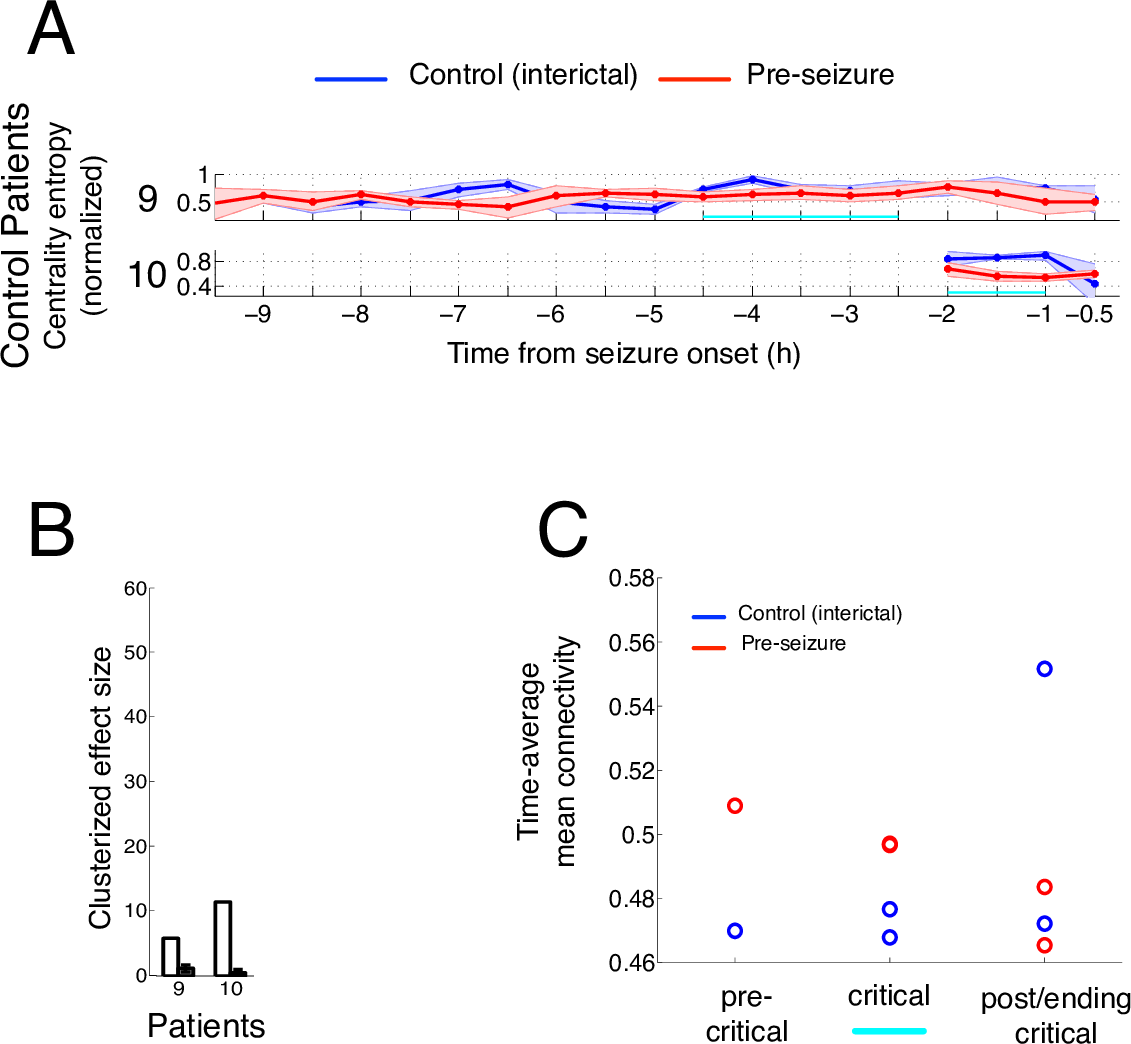

Supplement: S3 Fig — Time-dependent network state variability and functional connectivity for control patients. (A) Average normalized—to the (0, 1) range—centrality entropy for the control patients (n = 2) during a preseizure period (in red, 9.5 h before the first seizure) and a control period (in blue, 9.5 h from the preceding day). Averages were computed over time in nonoverlapping windows of 15 entropy samples each (total of 30 min) during both periods. Each entropy sample was computed in a smaller window of 200 subsamples (120 s). Curves represent the sequence of centrality entropy mean values, and error bars represent ± 1 SD. In cyan, the sequence of consecutive time steps lying in a significant clusterized difference (randomization test, P < 0.01). (B) Results for the cluster-based significance test. White bars show the value of the cluster-based statistic. Grey bars show the average across all surrogate statistic values. Error bars indicate ± 1 SD. (C) Time-average mean functional connectivity per patient (n = 2) along 3 consecutive subperiods of interest during preseizure and control periods. The first subperiod (precritical) comprises intervals prior to the significant cluster, the intermediate subperiod (critical) comprises intervals within the cluster, and the last subperiod (postcritical) comprises postcluster intervals. Underlying numerical values can be found in S7 Data. (TIF) [file pbio.2002580.s021.tif]

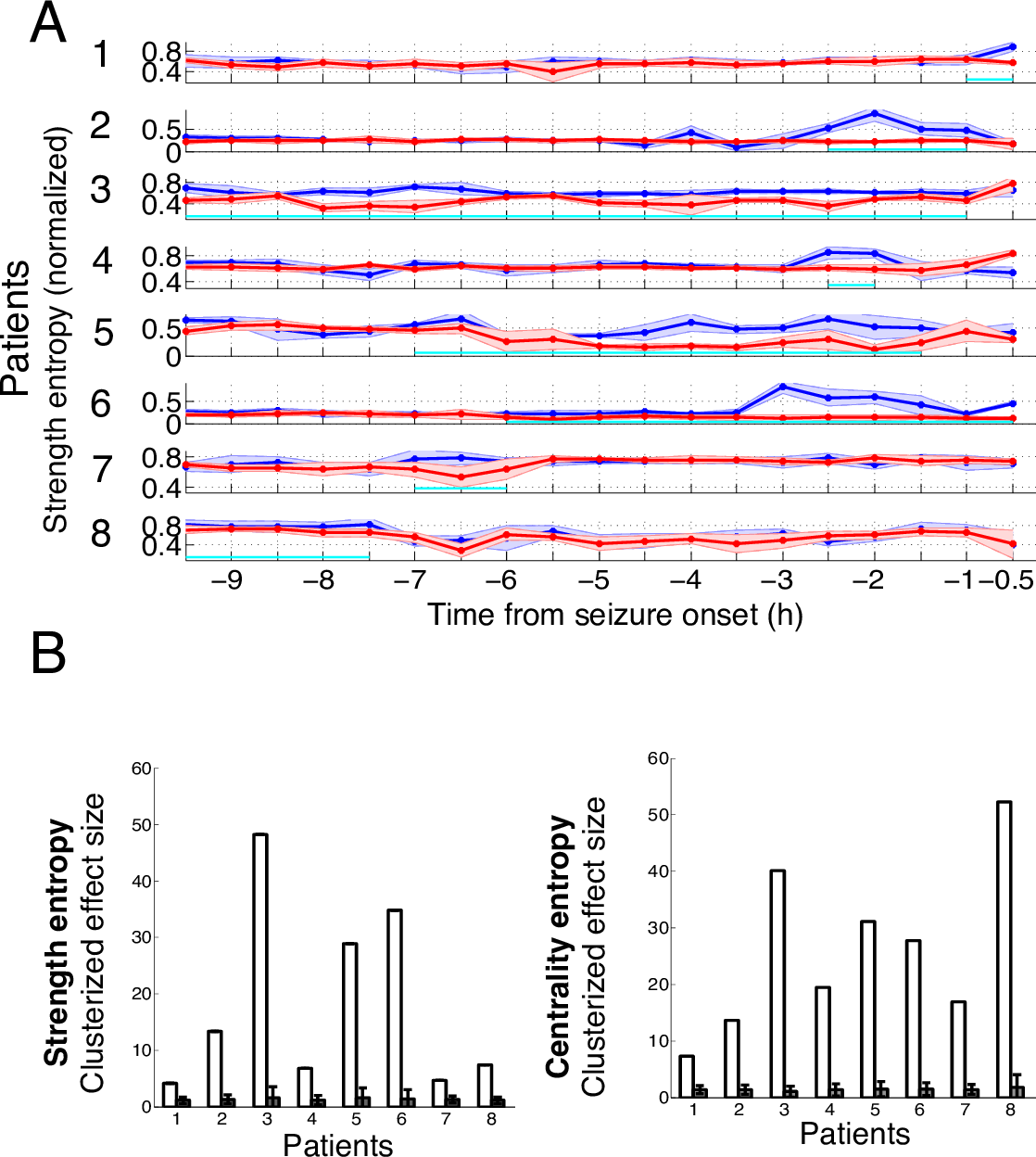

Supplement: S4 Fig — (A) Strength entropy curves for the control period (blue) and the preseizure period (red) are shown for every patient for 9.25 h preceding seizure onset time in a similar fashion as in Fig 2A. In cyan, the sequence of consecutive time steps lying in a significant clusterized difference (cluster-based randomization test, P < 0.01). (B) Results for the cluster-based significance test using the strength entropy (left) and the eigenvector centrality (right) to compute the multivariate entropies. White bars show the value of the cluster-based statistic. Grey bars show the average across all surrogate statistic values. Error bars indicate ± 1 SD. Underlying numerical values can be found in S8 Data. (TIF) [file pbio.2002580.s022.tif]

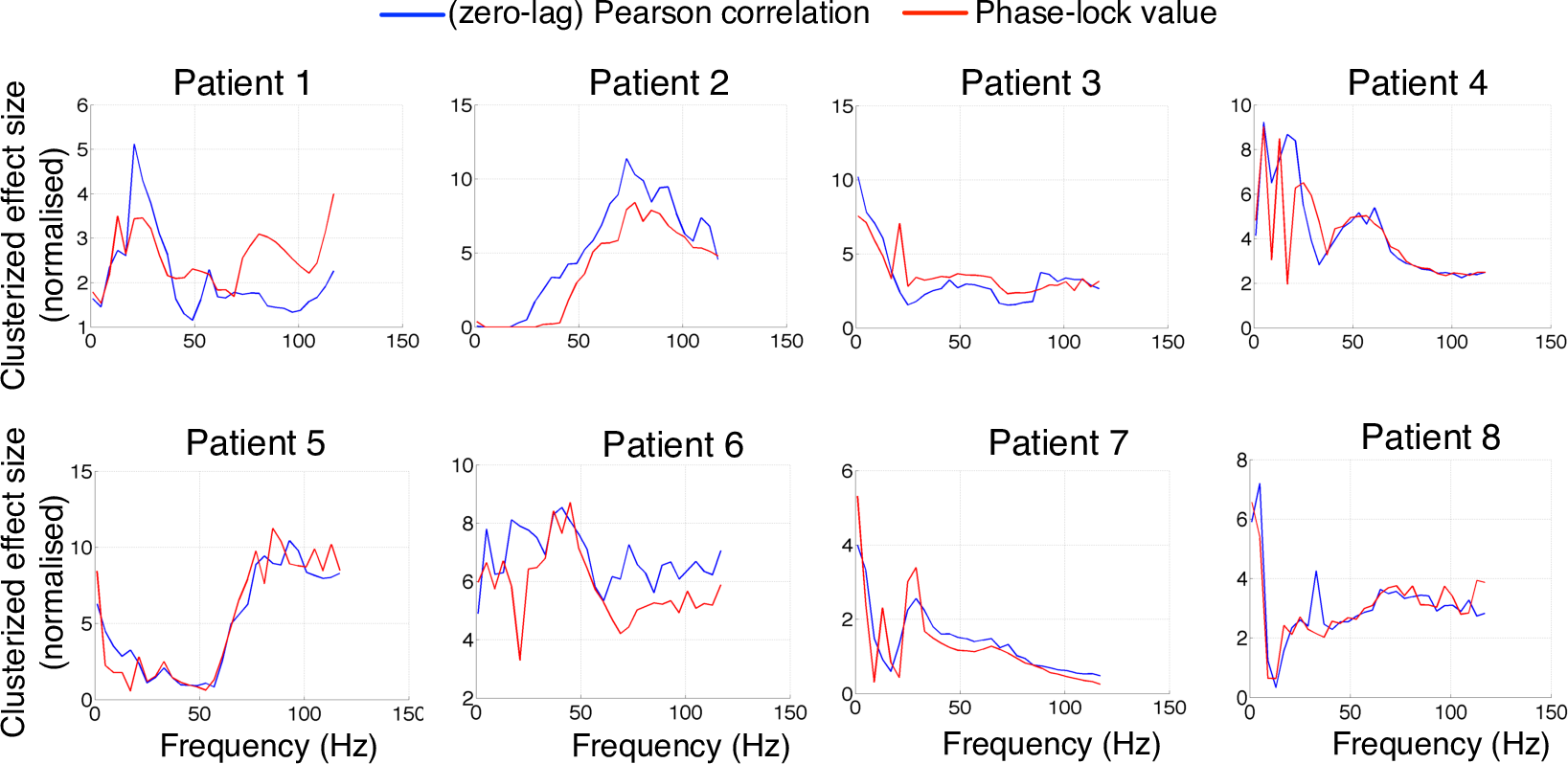

Supplement: S5 Fig — Cluster-based statistic of the nonparametric test (Fig 2A) computed for eigenvector centrality sequences based on Pearson correlation (original, blue) and phase-locking value (red) on frequency narrow bands from 1 to 120 Hz. Underlying numerical values can be found in S9 Data. (TIF) [file pbio.2002580.s023.tif]

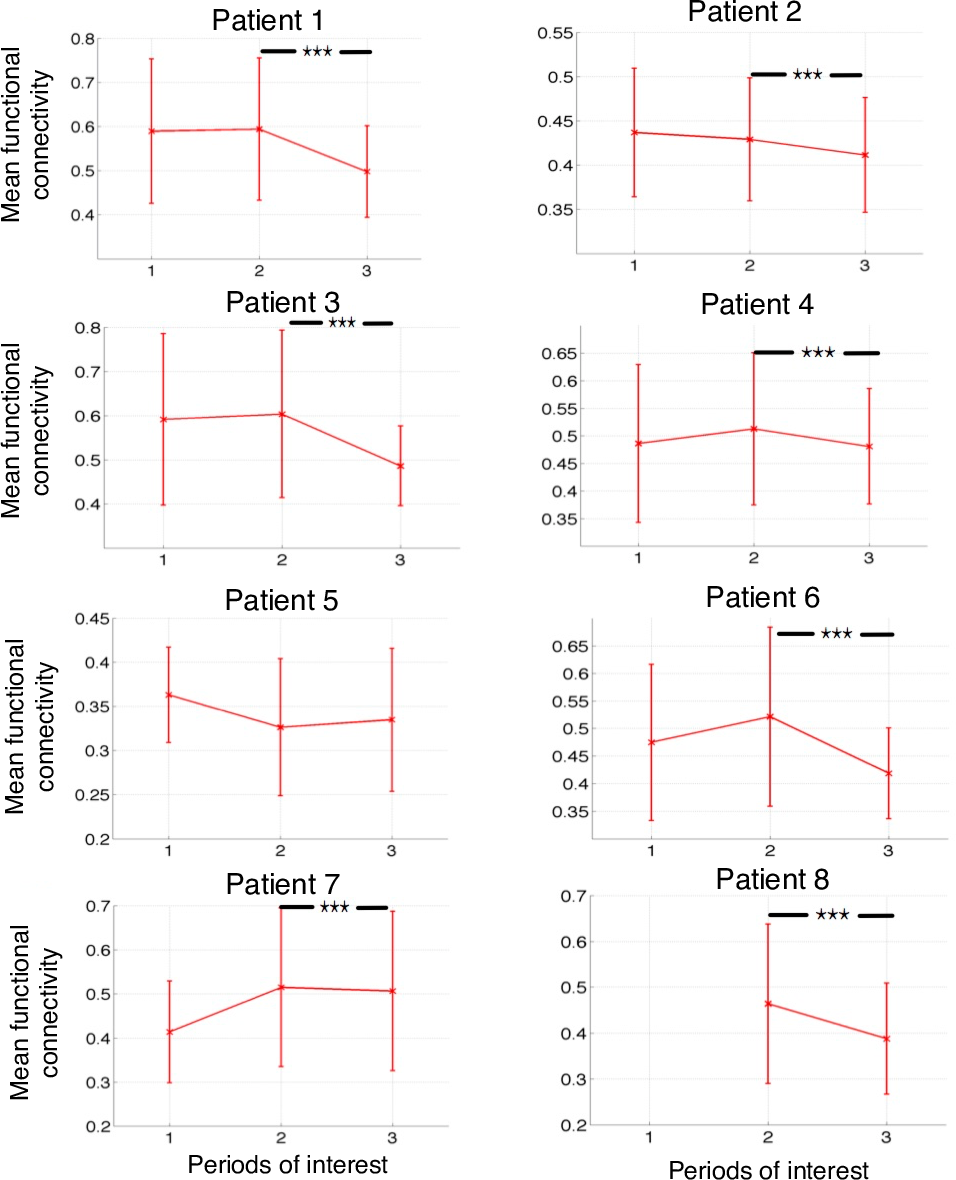

Supplement: S6 Fig — Time-average mean functional connectivity computed for each patient in 3 periods of interest for 9.25 h preceding the seizure onset time: the precritical phase (1), the critical phase (2), and the postcritical phase (3). In patients 1, 5, and 8, the last interval of the critical phase was considered to be in (3). Patient 8 did not present any interval before the critical phase in the period considered here. Error bars denote the SD across time samples. Stars denote that the decrease was significant in 7 out of 8 patients (P < 0.01, paired t test). Underlying numerical values can be found in S10 Data. (TIF) [file pbio.2002580.s024.tif]

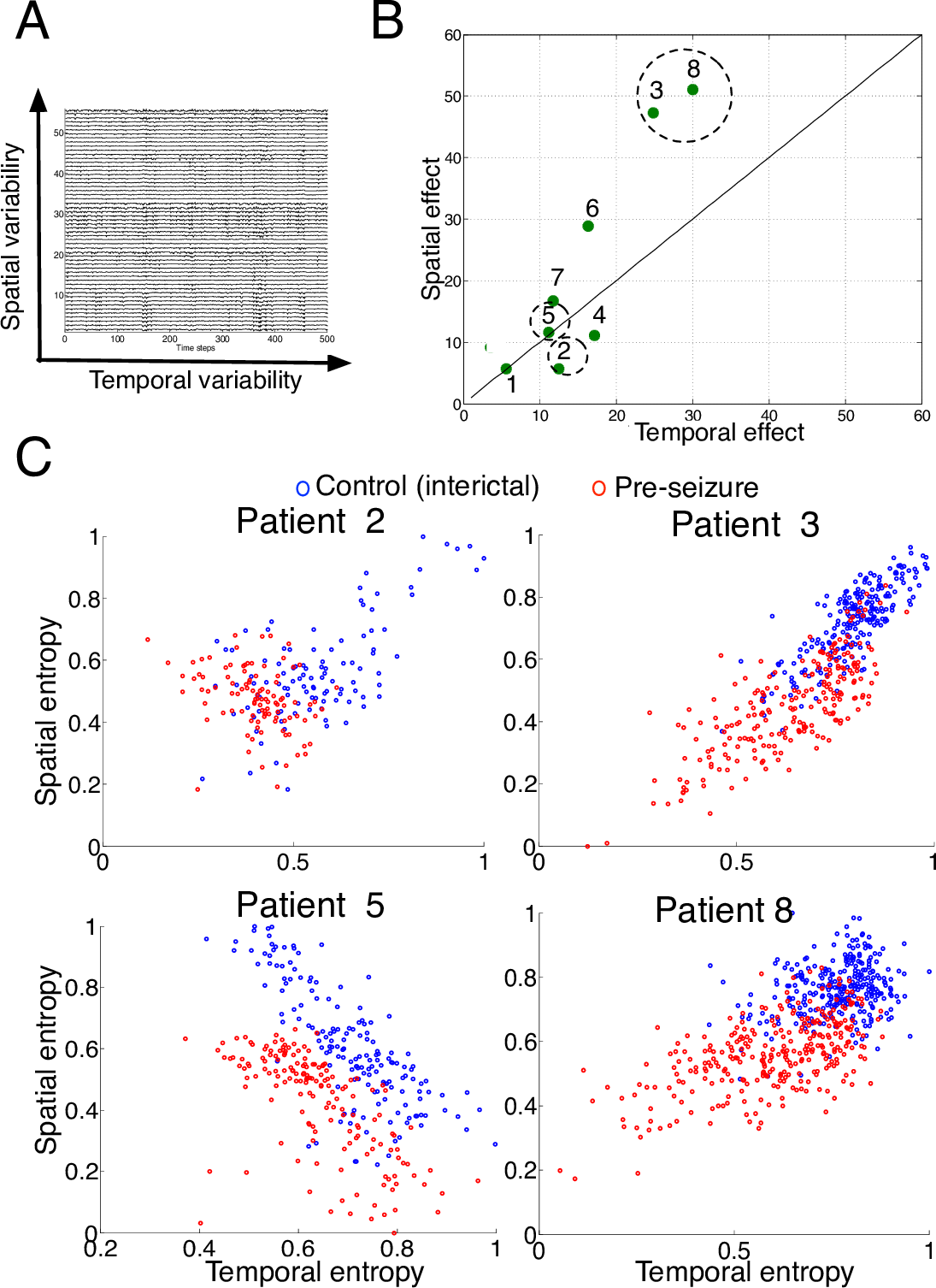

Supplement: S7 Fig — (A) Schematic representation of the 2 sources of variability in a set of simultaneous time series. (B) For every main patient, green dots representing pairs of statistic values (“temporal/spatial effects”) obtained from repeating the clusterized effect-size test (Fig 2A) with the spatial and temporal entropy, respectively. The circled effect pairs are exemplified in C. (C) For exemplary patients 2, 3, 5, and 8, the figure shows the decomposition of the centrality entropy values into pairs of temporal and spatial entropy values. In blue, pairs of entropy values obtained from the control segment. In red, pairs of entropy values obtained from the preseizure period. Underlying numerical values can be found in S11 Data. (TIF) [file pbio.2002580.s025.tif]

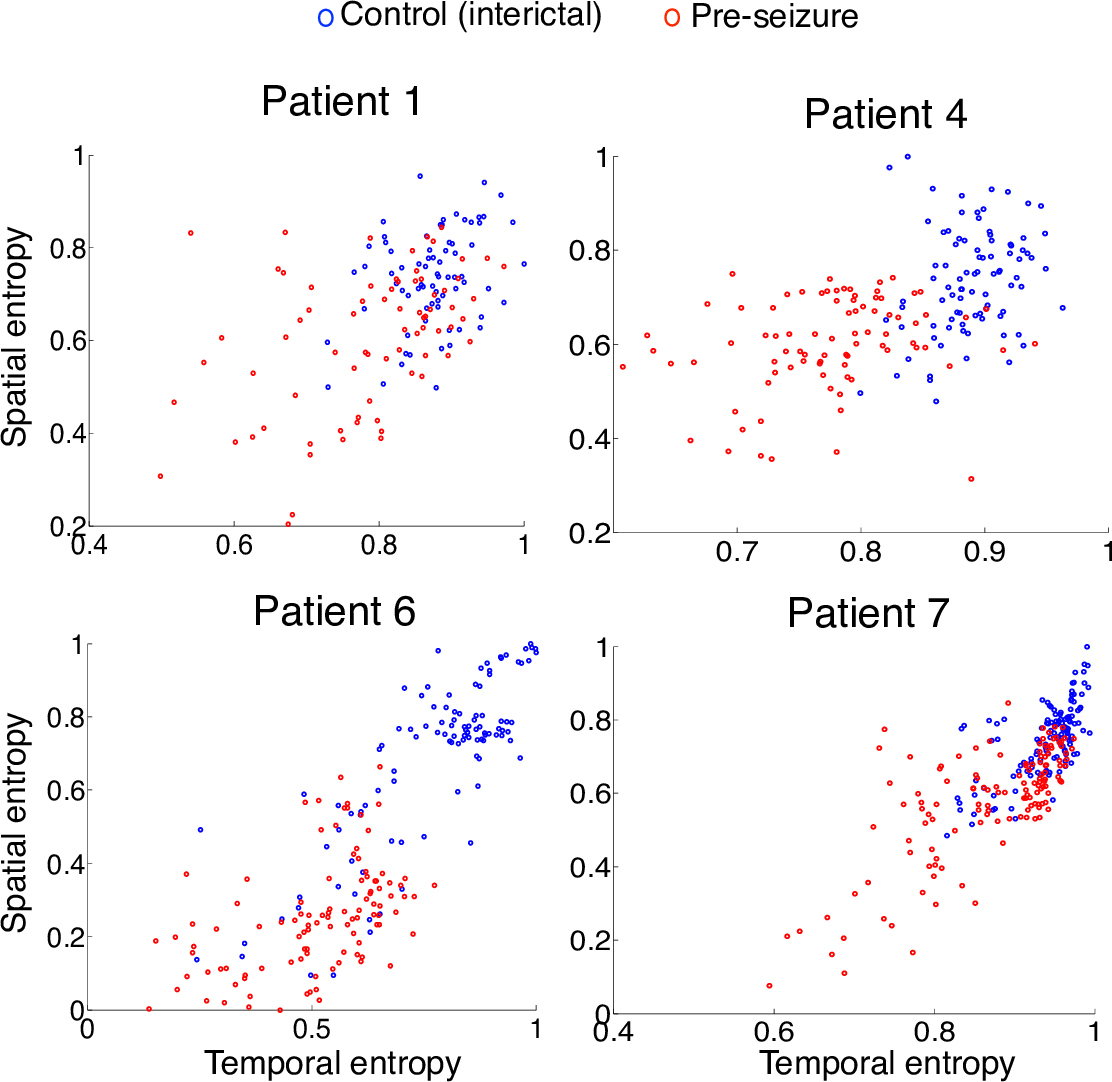

Supplement: S8 Fig — Centrality entropy decomposition of patients 1, 4, 6, and 7 into pairs of temporal and spatial entropy values. In blue, pairs of entropy values obtained from the control segment. In red, pairs of entropy values obtained from the preseizure period. Underlying numerical values can be found in S12 Data. (TIF) [file pbio.2002580.s026.tif]

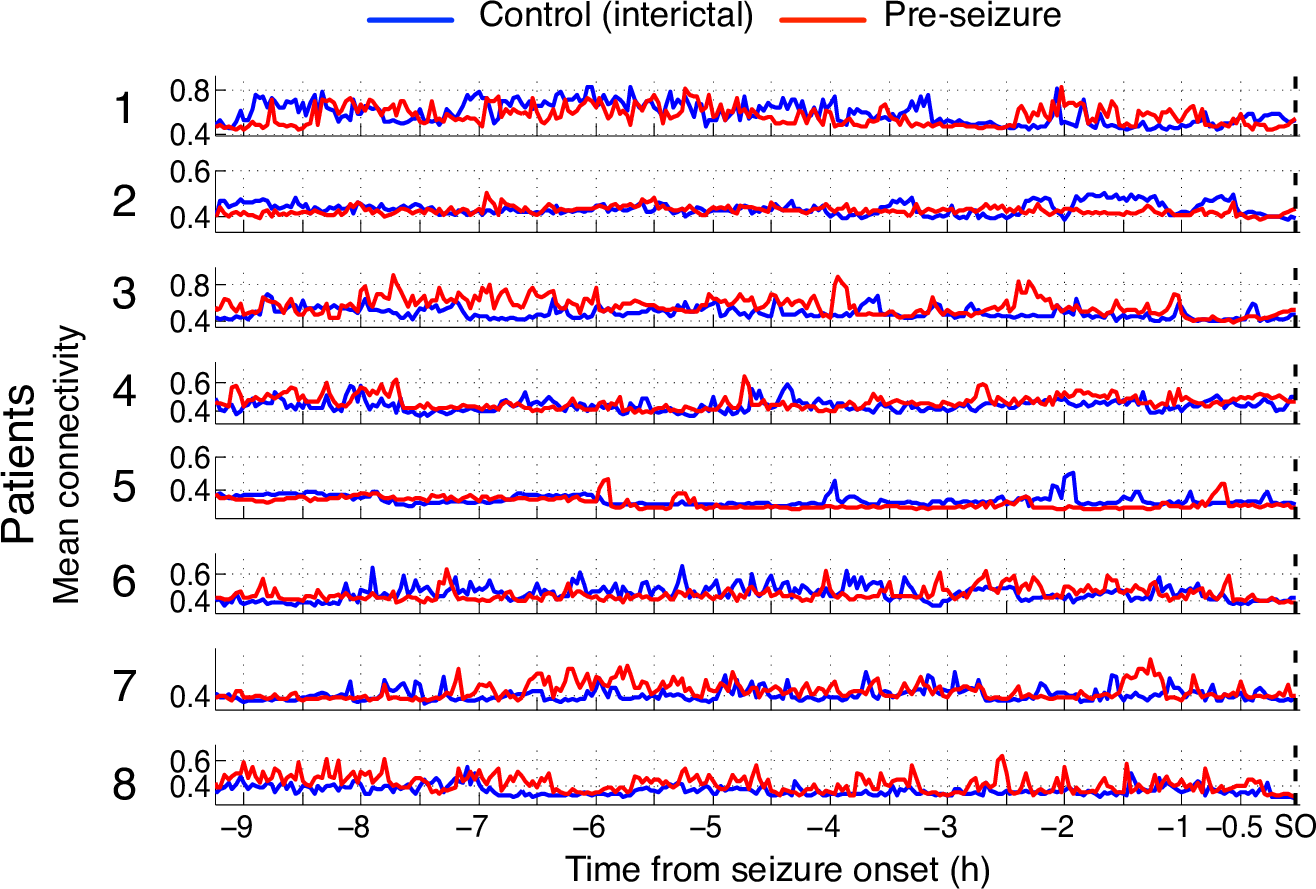

Supplement: S9 Fig — Time-average mean connectivity curves for the main patients for 9.25 h preceding seizure onset time. The mean connectivity was computed over all recording pairs in consecutive and nonoverlapping 0.6 s windows (300 samples). The time average was performed at the same time scale of the centrality entropy, i.e., 120 s (200 samples). In blue, curve corresponding to the control period. In red, curve corresponding to the preseizure period. Underlying numerical values can be found in S13 Data. (TIF) [file pbio.2002580.s027.tif]

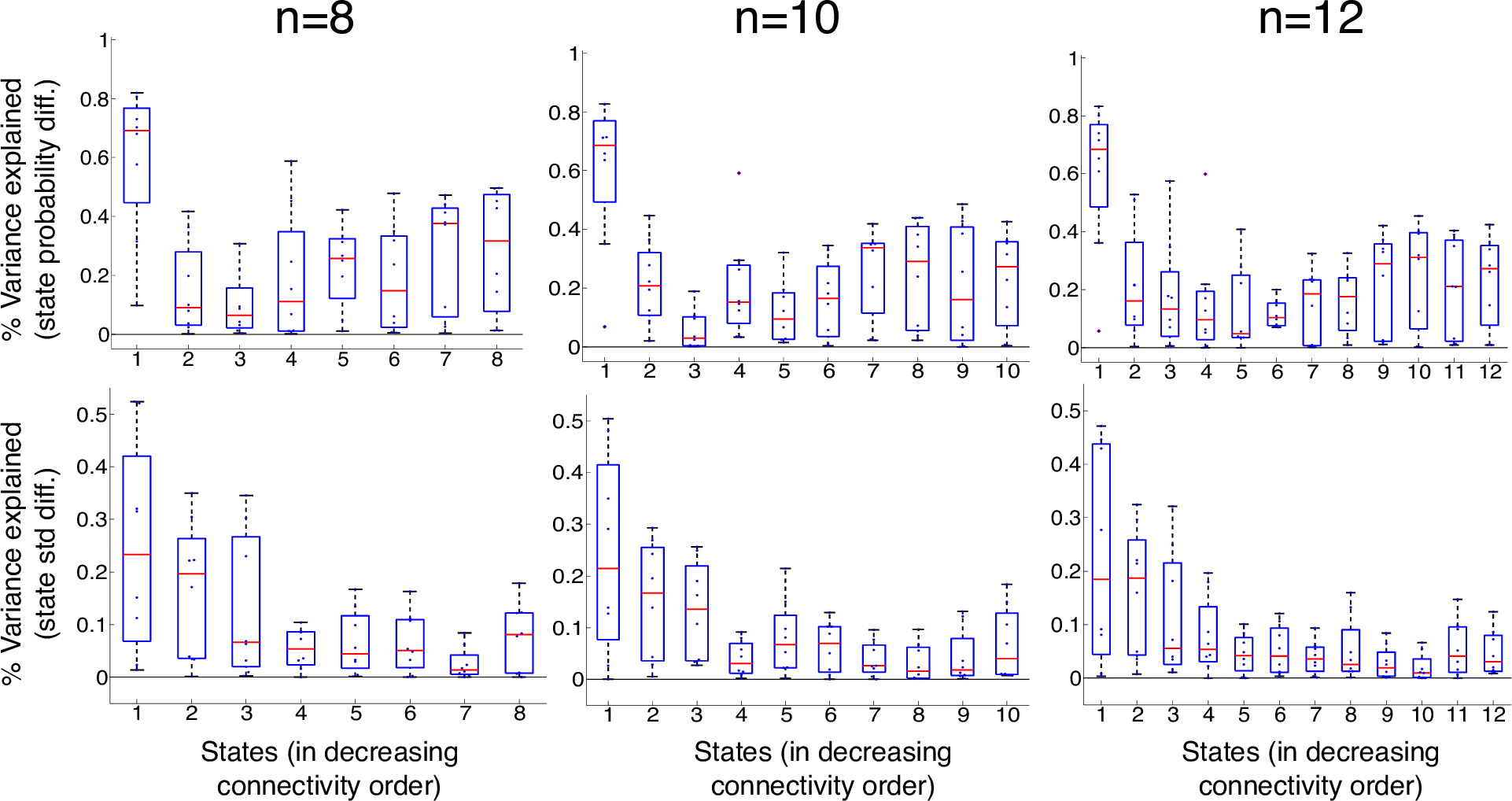

Supplement: S10 Fig — Variance explained by state probability and state homogeneity (“state SD”) differences in the crossperiod regression per patient of Fig 3B. when using n = 8 (left), n = 10 (center) discretized states and the original discretization (n = 12) shown in Fig 3C. (left). In the 3 cases, discretized states were sorted along the horizontal axis in mean connectivity decreasing order for each patient. For each sorted state, boxplots show the distribution of the coefficient of determination (% variance explained) across patients. Underlying numerical values can be found in S14 Data. (TIF) [file pbio.2002580.s028.tif]

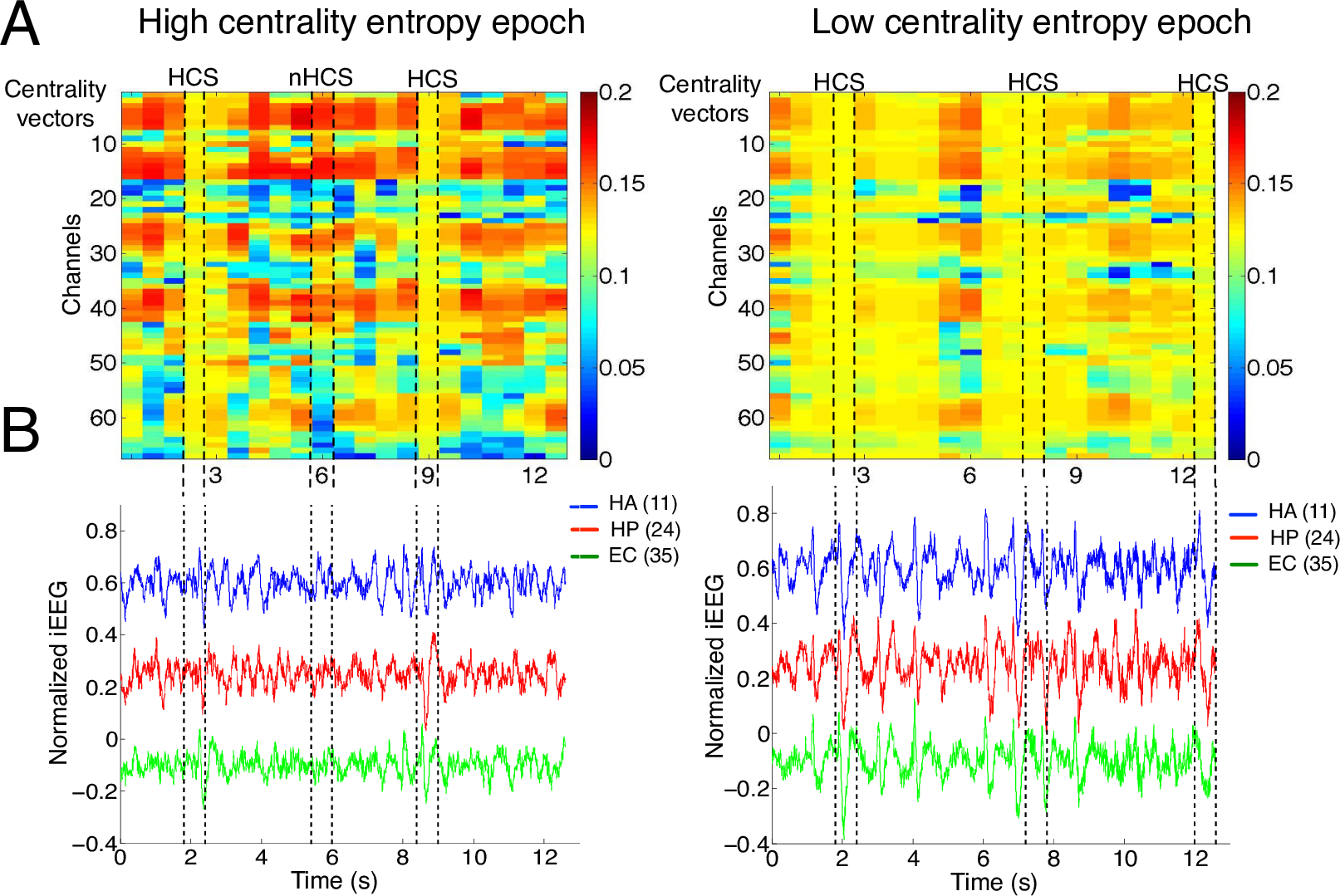

Supplement: S11 Fig — (A) Sequence of 21 (12.6 s) consecutive centrality eigenvectors extracted from an epoch with high-centrality entropy values (left) and from an epoch with low-centrality entropy values (right) in the preseizure period of patient 3. Color intensity (blue = lowest, red = highest) represents centrality values. Horizontal dashed lines delimit HCSs (homogenous yellow strips) and nHCSs (heterogeneous strips) per epoch of 0.6 s duration. (B) Normalized and voltage-shifted (for visualization purposes) iEEG recordings in HA (channel 11, blue), HP (channel 24, red), and EC (channel 35, green) in the high-centrality entropy (left) and low-centrality entropy (right) epoch. Horizontal dashed lines delimit the iEEG recording segments corresponding to the previous HCSs and nHCSs. Underlying numerical values can be found in S15 Data. EC, entorhinal cortex; HA, anterior hippocampus; HCS, high-connectivity state; HP, posterior hippocampus; iEEG, intracranial electroencephalography; nHCS, non–high-connectivity state. (TIF) [file pbio.2002580.s029.tif]

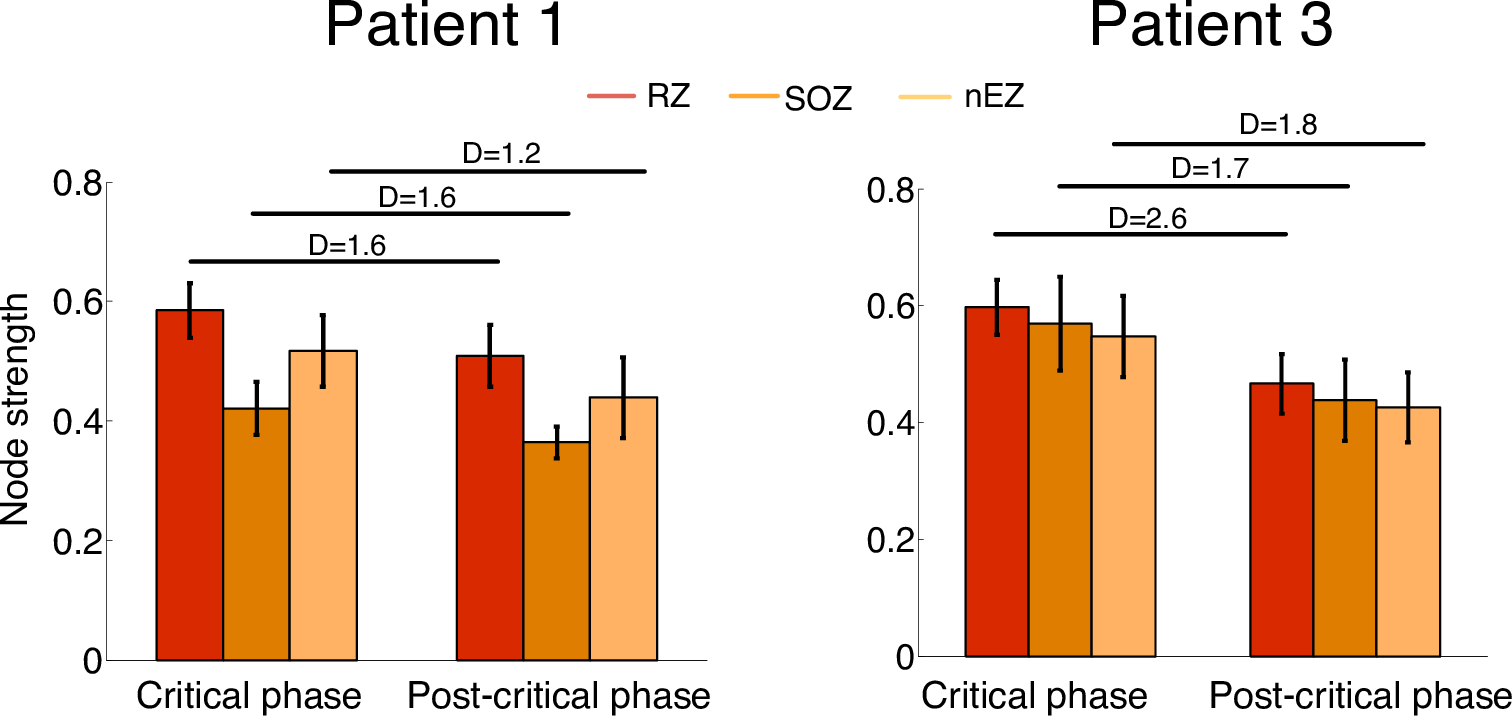

Supplement: S12 Fig — For patients 1 and 3 (patients with best postsurgical outcome after resection), bars showing the site-average connectivity strength of the RZ, SOZ, and remaining sites (nEZ) during the critical phase (left) and postcritical phase (right) in the preseizure period. Strength samples were computed for each site by performing averages over time samples (0.6 s) during each phase. Effect sizes were reported for each sites’ group by computing Cohen’s d across both phases. Underlying numerical values can be found in S16 Data. nEZ, nonepileptogenic zone; RZ, resected zone; SOZ, seizure-onset zone. (TIF) [file pbio.2002580.s030.tif]

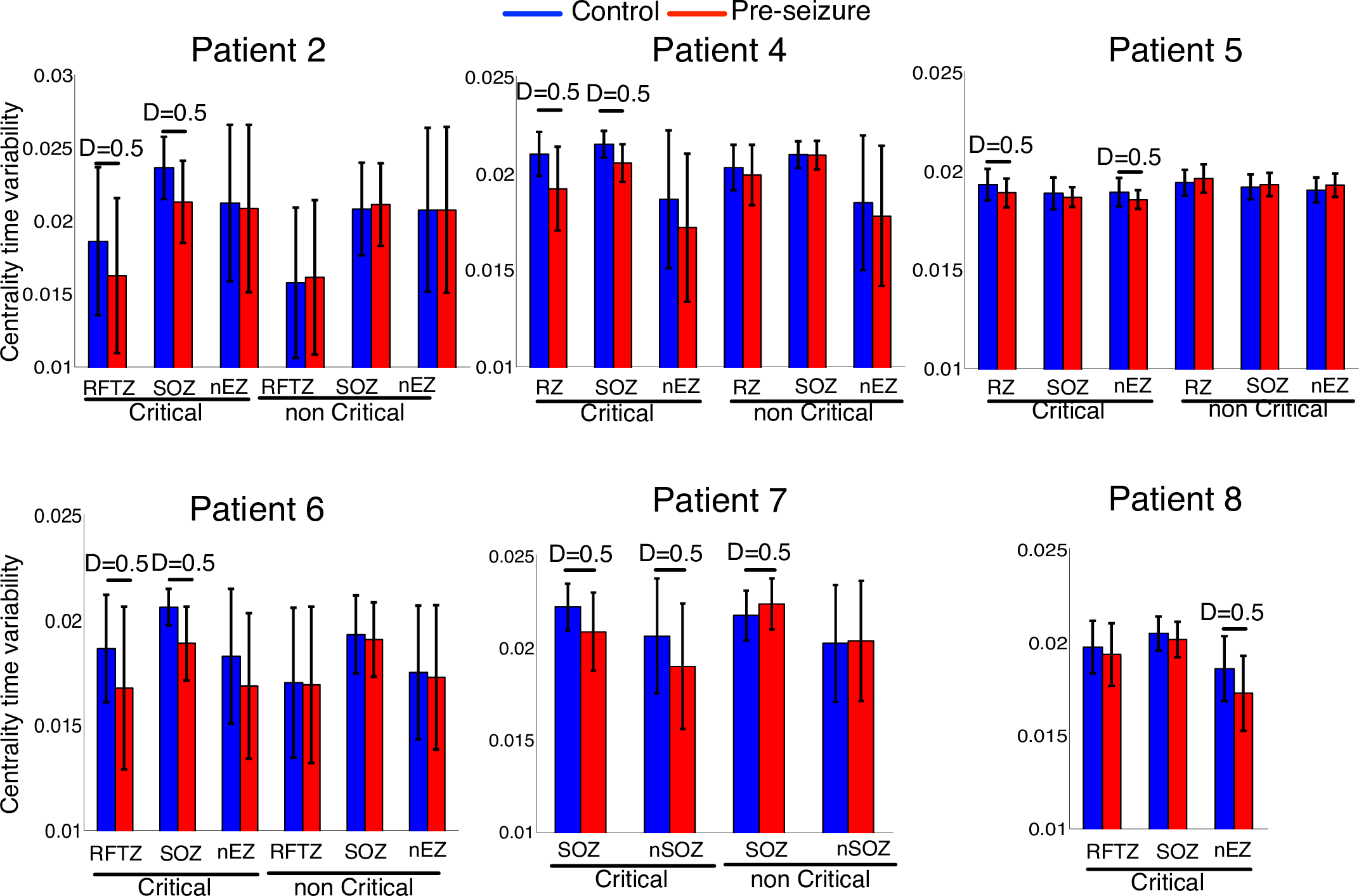

Supplement: S13 Fig — For patients 2, 4, and 5–8, crossperiod comparison (control in blue, preseizure in red) of sites’ centrality variability averaged over RZ, SOZ, and nEZ inside (critical, left) and outside (noncritical, right) the estimated critical phase whenever it was possible. Each sample per recording site was computed by performing an average (across critical and noncritical phases) of the centrality’s temporal SD measured in consecutive and nonoverlapping time windows of 120 s (200 samples). Sizes of significant effects (paired t test, P < 0.05) equal or larger to 0.5 were reported using Cohen’s d and approximated to the first decimal. In all subfigures, error bars represent ± 1 SD. Underlying numerical values can be found in S17 Data. nEZ, nonepileptogenic zone; RZ, resected zone; SOZ, seizure-onset zone. (TIF) [file pbio.2002580.s031.tif]

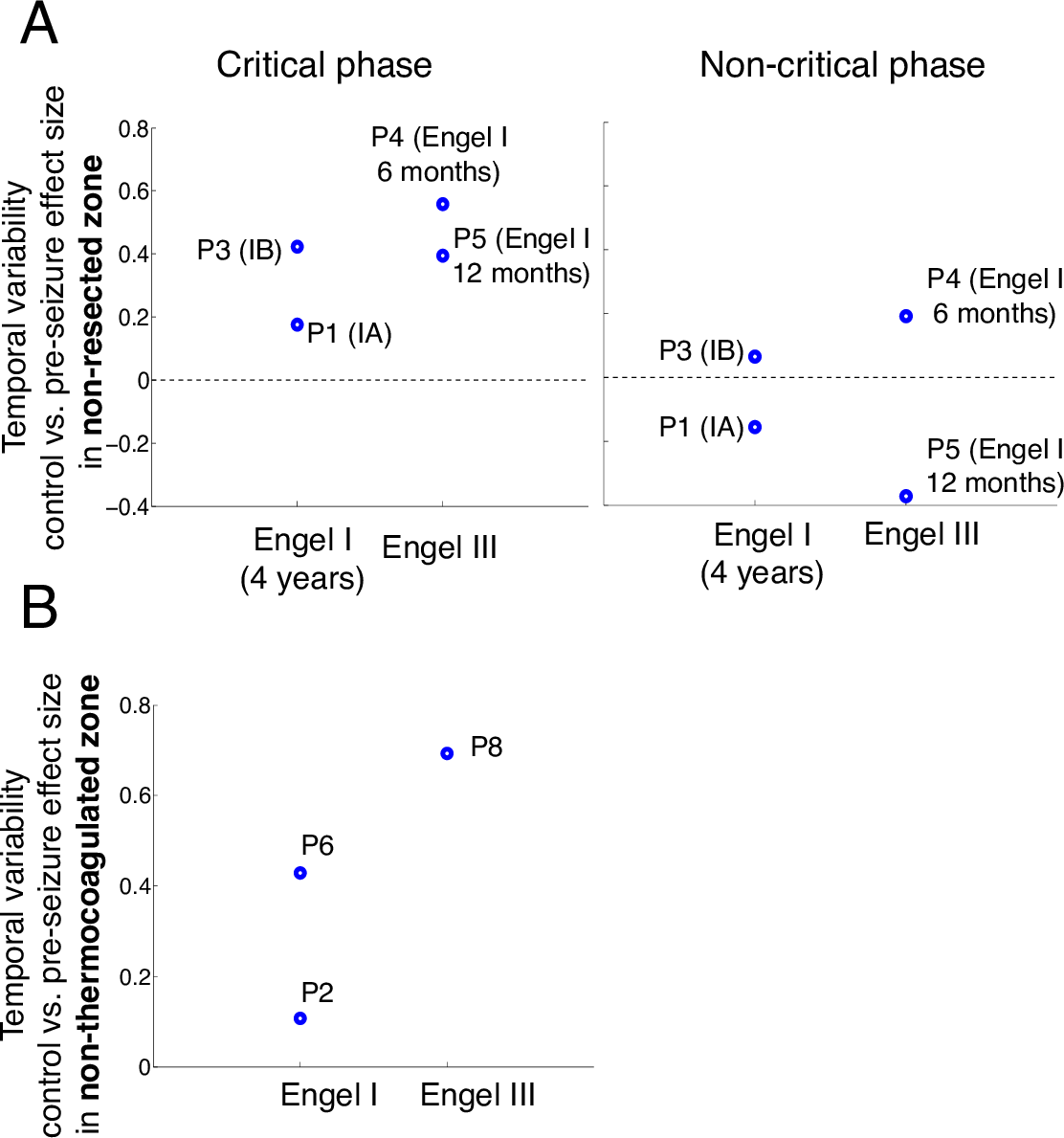

Supplement: S14 Fig — For the main patients (n = 7) who underwent either surgery or RFTC, dots represent the effect size (Cohen’s d) of preseizure changes in the centrality time variability of nontreated regions (nonresected or nonablated) as a function of the postoperative outcome. (A) Results for surgical treatment (patients 1 and 3–5) inside (left) and outside (right) the critical phase. (B) Results for RFTC treatment (patients 2, 6, and 7) inside the critical phase. Underlying numerical values can be found in S18 Data. RFTC, radiofrequency thermocoagulation. (TIF) [file pbio.2002580.s032.tif]
